# Exploring Pandora's Box: Potential and Pitfalls of Low Coverage Genome Surveys for Evolutionary Biology

**DOI:** 10.1371/journal.pone.0049202

**Published:** 2012-11-21

**Authors:** Florian Leese, Philipp Brand, Andrey Rozenberg, Christoph Mayer, Shobhit Agrawal, Johannes Dambach, Lars Dietz, Jana S. Doemel, William P. Goodall-Copstake, Christoph Held, Jennifer A. Jackson, Kathrin P. Lampert, Katrin Linse, Jan N. Macher, Jennifer Nolzen, Michael J. Raupach, Nicole T. Rivera, Christoph D. Schubart, Sebastian Striewski, Ralph Tollrian, Chester J. Sands

**Affiliations:** 1 Ruhr University Bochum, Department of Animal Ecology, Evolution and Biodiversity, Bochum, Germany; 2 British Antarctic Survey, High Cross, Madingley Road, Cambridge, United Kingdom; 3 Alfred Wegener Institute for Polar and Marine Research, Functional Ecology, Bremerhaven, Germany; 4 Zoologisches Forschungsmuseum Alexander Koenig, Bonn, Germany; 5 Senckenberg am Meer, German Center for Marine Biodiversity Research, Molecular Taxonomy Group, Wilhelmshaven, Germany; 6 University of Regensburg, Biologie 1, Department of Evolution, Behavior and Genetics, Regensburg, Germany; Onderstepoort Veterinary Institute, South Africa

## Abstract

High throughput sequencing technologies are revolutionizing genetic research. With this “rise of the machines”, genomic sequences can be obtained even for unknown genomes within a short time and for reasonable costs. This has enabled evolutionary biologists studying genetically unexplored species to identify molecular markers or genomic regions of interest (e.g. micro- and minisatellites, mitochondrial and nuclear genes) by sequencing only a fraction of the genome. However, when using such datasets from non-model species, it is possible that DNA from non-target contaminant species such as bacteria, viruses, fungi, or other eukaryotic organisms may complicate the interpretation of the results. In this study we analysed 14 genomic pyrosequencing libraries of aquatic non-model taxa from four major evolutionary lineages. We quantified the amount of suitable micro- and minisatellites, mitochondrial genomes, known nuclear genes and transposable elements and searched for contamination from various sources using bioinformatic approaches. Our results show that in all sequence libraries with estimated coverage of about 0.02–25%, many appropriate micro- and minisatellites, mitochondrial gene sequences and nuclear genes from different KEGG (Kyoto Encyclopedia of Genes and Genomes) pathways could be identified and characterized. These can serve as markers for phylogenetic and population genetic analyses. A central finding of our study is that several genomic libraries suffered from different biases owing to non-target DNA or mobile elements. In particular, viruses, bacteria or eukaryote endosymbionts contributed significantly (up to 10%) to some of the libraries analysed. If not identified as such, genetic markers developed from high-throughput sequencing data for non-model organisms may bias evolutionary studies or fail completely in experimental tests. In conclusion, our study demonstrates the enormous potential of low-coverage genome survey sequences and suggests bioinformatic analysis workflows. The results also advise a more sophisticated filtering for problematic sequences and non-target genome sequences prior to developing markers.

## Introduction

Recent advances in high throughput sequencing technologies have caused a paradigm shift in molecular evolutionary biology [1]. Whereas traditionally the analysis of many markers was a costly and tedious task and restricted mainly to genetic model organisms, it is now possible to screen large proportions of previously unexplored genomes with high-throughput sequencing methods almost as easily as known genomes. This hugely facilitates ecological and evolutionary studies [2] and promises to overcome the statistical pitfalls associated with still often-used single marker studies (see [3] for discussion). With the “rise of the machines” [4], novel methodological possibilities are provided for addressing questions at both micro- (e.g. [5], [6]) and macroevolutionary levels (e.g. [7], [8]). The basic principle common to both is that the genomic regions identified for marker development and analysis should be informative enough to answer the biological question under study. For microevolutionary questions, genomic regions with high variability at the population level are important [9], whereas in studies addressing old speciation events markers need to be less variable to avoid problematic homoplasious signals (e.g. [10]). For microevolutionary studies, microsatellites and minisatellites often represent the marker system of choice due to their high variability [9], [11], [12]. Here, with high-throughput sequencing, the straightforward sequencing of enriched and non-enriched libraries on fractions of 454 plates can provide a good solution when searching for microsatellite markers [13]–[16] (for a review see [4], [17], [18]).

For studies aiming to investigate recent divergence processes between species, mitochondrial genes were and still are often the first choice [19]. Most of the mitochondrial genes evolve comparatively fast and have the advantage of being haploid and abundant in cells. If evolutionary events that date back many millions of years are the central theme of a study, the analysis of more conservative (slow evolving) regions is appropriate to avoid too many multiple substitutions overwriting the meaningful signal. Typical regions for phylogenetic questions are the conserved core regions of the nuclear multicopy rRNA genes [20].

With high-throughput sequencing, large sets of expressed sequence tags (ESTs) or specifically targeted nuclear genes can be amplified and compared among taxa [7], [8], [21]. An interesting point in this respect is that with deep sequencing of nuclear or mitochondrial genomes it is not only the sequence variation between homologous loci that can be used as phylogenetic information, but also the genome morphology, i.e. the order and organisation of the mitochondrial genes [22]–[24]. Mitochondrial genome sequencing by traditional methods, such as primer walking strategies or the use of conserved primers for long-range PCR, are time-consuming and have a limited success rate whereas high-throughput sequencing approaches can greatly facilitate development of complete or nearly complete mitochondrial genomes [25]–[27].

In many published high-throughput sequencing studies, the sequence libraries are only partially explored, focussing on a particular set (certain protein coding genes) or type (microsatellites) of markers and often neglect potential pitfalls of high-throughput data. In particular, contamination of genomic libraries by bacteria, viruses or symbionts, by human material or cross-contamination is a well known problem (e.g. [28], [29]). Such contamination can bias subsequent evolutionary analyses leading to erroneous conclusions (e.g. [30]–[32]). Therefore, the detection and removal of contaminant sequences is important prior to downstream analysis. Bioinformatic tools that aid in the process of identifying contamination by heuristic comparisons of query sequences against reference databases, such as BLAST [33], BLAST+ [34] and BLAT [35], or programs that map the new sequences against reference genomes such as BWA [36], BWA-SW [37] or SSAHA [38], can further speed up and improve the process of identifying and removing contaminant sequences from the genomic libraries (see [29] for a comparison of programs on metagenomic datasets).

The current study builds upon the first studies that have documented the potential of low coverage genome surveys, which analyse only a part of the whole genome, for evolutionary inferences (e.g. [25], [39]). With the goal of widening the scope of low-coverage genome survey data, we explore their use not only for one marker type, but for 1) micro- and minisatellites, 2) mitochondrial genes and genomes, and 3) for nuclear genes (protein-coding genes, rRNA genes, transposable elements). Moreover, we demonstrate that several mid- to small budget labs can tap into the potential of high-throughput sequencing by sharing costs and thus maximizing output. A central objective of this study was to analyse the data for possible contamination by viruses, bacteria or endosymbionts. Our high-throughput libraries originate from genetic non-model species and may thus be more representative of the majority of exploratory biological studies. Our results from 14 non-enriched genomic libraries show that low-coverage genome surveys of non-model organisms can yield many informative genetic markers besides microsatellites. However, we also identify significant contributions of intragenomic, intra- and extracellular DNA contamination in several libraries that, if not systematically identified and removed using bioinformatic techniques, can lead to erroneous conclusions about the evolutionary processes under study.

## Materials and Methods

### Species investigated

We analysed 14 genomic libraries of species from four different animal phyla (1 cnidarian, 9 arthropods, 3 molluscs, 1 echinoderm, see Table 1). Furthermore, we also analysed two libraries enriched for microsatellites according to the protocol by Leese et al. [40] from the mollusc *Lissarca notorcadensis* and the asteroid echinoderm *Odontaster validus* (Table 1).

**Table 1:** Species analysed in this study and characteristics of the libraries.

Library	Taxonomy	Plate	Library type	Number of reads	Average read length, bp	Sum of read lengths, bp	Number of contigs	Sum of contig lengths, bp	Average contig length, bp
**Cnidaria**									
*Favia fragum* (Esper)	Anthozoa: Scleractinia	1	genomic	96,040	376.6	34,055,442	69,405	27,520,221	396.5
**Arthropoda**									
*Austropallene cornigera* (Möbius)	Pycnogonida	1	genomic	73,557	293.0	20,396,151	40,883	13,973,404	341.8
*Colossendeis megalonyx* Hoek	Pycnogonida	1	genomic	100,719	259.4	25,499,956	57,425	17,539,519	305.4
*Pallenopsis patagonica* (Hoek)	Pycnogonida	2	genomic	134,846	325.5	41,741,628	62,753	25,378,904	404.4
*Uristes adarei* (Walker)	Malacostraca: Amphipoda	1	genomic	68,047	211.5	15,482,430	43,336	10,580,572	244.2
*Euphausia superba* Dana	Malacostraca: Euphausiacea	1	genomic	49,802	247.6	12,098,817	42,256	10,868,476	257.2
*Nematocarcinus lanceopes* Bate	Malacostraca: Decapoda	2	genomic	168,267	250.3	43,343,686	79,740	25,343,246	317.8
*Hyas araneus* (Linnaeus)	Malacostraca: Decapoda	1	genomic	175,098	244.8	44,890,134	93,050	28,037,451	301.3
*Metopaulias depressus* Rathbun	Malacostraca: Decapoda	2	genomic	186,890	265.5	55,152,741	63,040	26,186,278	415.4
*Sericostoma personatum* (Kirby & Spence)	Hexapoda: Trichoptera	2	genomic	253,210	336.3	78,747,514	139,237	53,927,755	387.3
**Mollusca**									
*Lepetodrilus* sp. nov.	Gastropoda: Vetigastropoda	2	genomic	339,640	330.7	105,577,603	178,788	69,304,964	387.6
*Limatula hodgsoni* (Smith)	Bivalvia: Limoida	2	genomic	168,113	241.1	39,015,159	105,801	28,438,377	268.8
*Arctica islandica* (Linnaeus)	Bivalvia: Veneroida	1	genomic	71,385	308.3	20,577,244	54,266	17,790,671	327.8
*Lissarca notorcadensis* Melvill & Standen	Bivalvia: Arcoida	1	enriched	205,905	194.7	46,847,086	84,498	17,059,967	201.9
**Echinodermata**									
*Odontaster validus* Koehler	Asteroidea	1	enriched	183,166	200.5	39,311,172	86,280	17,972,482	208.3
*Gorgonocephalus chilensis* (Philippi)	Ophiuroidea	1	genomic	60,181	330.8	18,452,499	39,809	14,681,395	368.8

The number of reads and number of Mbp refers to the unassembled raw data, whereas the number of contigs refers to the number of unique sequences after assembly with MIRA.

### DNA extraction and sequencing

For DNA isolation, specimen tissue was extracted under clean conditions in the lab to avoid contamination. For each genomic library, DNA was extracted (see Supporting information S1) and 5 µg of genomic DNA sent to Macrogen Inc. (Seoul, South Korea) for library preparation. Individually-tagged libraries were analysed on two full 454 plates on a GS-FLX sequencer (Roche) (Table 1).

### Assembly

From the raw sequence files, FASTA, quality and trace information files were extracted using the sff_extract v. 0.2.8 python script [41]. Sequence tags of the reads were clipped. The processed raw data were assembled using MIRA version 3.2.1.5 [42] using the 454 default settings of the “de novo, genome, accurate, 454” mode with two modifications after several tests: The parameter AL:mrs was set to 85 (default 70), which is the minimum percentage similarity of two overlapping sequences to be assembled, The parameter AS:mrpc was set to 2, i.e. at least two reads (and not five or more as usual in higher coverage situations) were needed to create a contig (see results for full explanation of the parameters). The MIRA assembler was chosen since it has unique features such as chimera clipping, repeat masking and a very flexible algorithm that can be adjusted to the specific 454 low-coverage data. The quality of the assemblies was visually inspected using Geneious 5.4.6 [43]. To aid further analyses the contigs were uploaded into a custom MySQL database (MySQL-server v. 5.1.44) [44]. All of the filtering steps and the final dataset production were performed in the database using SQL-commands.

The Animal Genome Size Database [45] was used as a primary resource to obtain genome sizes to compute approximate genomic coverages for the libraries. We selected the closest relatives to our target species from the database for comparison. Especially in cases for which no closely related species were found in the database, this approximation is to be treated with caution. For the genome size estimates of the Antarctic krill (*Euphausia superba*) we used the recently published information on genome size ranges published by Jeffrey [46].

### Taxonomic and functional characterization of the contigs

In order to classify the contigs we performed a number of BLAST searches with different strategies (see below) [33]. The results were parsed and uploaded into the MySQL-database. We used the accession numbers from the BLAST hits to obtain the “definition” and “description” sections of the corresponding sequences as well as the associated taxonomic information using an in-house tool that retrieves this information automatically from the NCBI Entrez Utilities Web Service (see http://www.ncbi.nlm.nih.gov/entrez/query/static/esoap_help.html). These data were stored in the database and queried later for functional and taxonomic assignment; summary statistics and inputs for subsequent downstream processing were obtained.

For the purpose of taxonomic annotations, BLASTn searches with our sequences as queries against the whole nucleotide collection of NCBI sequences were performed on local servers. A conservative threshold e-value of ≤10^−12^ was used. Only the best hits were collected and stored in the MySQL database. These data were used to obtain information about non-eukaryotic sequences and sequences derived from known mobile elements (see section “transposable elements” below) and to produce “contamination-free” datasets in which these sequences were removed.

### Tandem repeat analysis

Micro- and minisatellites (1–6 basepairs (bp) and 7–50 bp motif length, respectively) were searched for in all contamination-free (see below) contigs and single reads with a minimum length of 100 bp. This tandem repeat search was performed using the software Phobos 3.3.12 [47]. Since different studies used different software and search criteria to find tandem repeats (see [48] for discussion) we applied three different parameter settings to compare the results with other studies. First, we used the search parameters used in a recent comparative study on micro- and minisatellites [48] (Phobos parameter settings –searchMode imperfect -u 1 -U 6 -g -5 -m -5 -s 12). In order to design primers for only the best loci, the results were filtered for 100% perfect microsatellites. Second, we applied the search criteria used by Santana et al. [15] to search for microsatellites (equivalent Phobos parameters –searchMode exact -u 1 -U 1 -s 11 for mononucleotide repeats and –searchMode exact -u 2 -U 6 –minLength_b 5 -s 8 for di- to hexanucleotide repeats). Third, we employed the search parameters used by Abdelkrim et al. [13] and Gardner et al. [4] (equivalent Phobos parameters –searchMode exact -u 2 -U 6 –minLength_b -s 8). With the exception of [48] these studies did not explicitly search for minisatellites. In this study we searched for minisatellites in the range of 7–50 bp motifs with the Phobos settings -u 7 -U 50 -R 30 -m -5 -g -5 -s 12 [48].

Since the aim of the study was to detect tandem repeats that could be used as genetic markers we performed a search for appropriate primers annealing to the respective flanking regions with Primer3 v. 2.3.4 [49]. The parameters were the default ones with the following modifications: PRIMER_MAX_NS_ACCEPTED = 1, PRIMER_PRODUCT_SIZE_RANGE = 100–300, PRIMER_PAIR_MAX_DIFF_TM = 8, PRIMER_MAX_POLY_X = 4, PRIMER_NUM_RETURN = 3 and all tandem repeats were masked with SEQUENCE_EXCLUDED_REGION. Further, all primer pairs were checked whether the respective regions had low complexity (“cryptic simplicity”). This simplicity test was performed with SIMPLE v. 5 [50], [51]. The parameters were as follows: sequence type ‘n’ (DNA/RNA), equal weights for mono- to tetranucleotide motifs, 50 random sequences, shuffle elements method, and (half-) window size of 4. From the maximum of three primer pairs queried we stored either the pair without signs of simplicity or just the best one if primers in all pairs were significantly simple. Following the recommendation by Meglecz et al. [52] we made a final stringency filtering retaining only single read contigs with appropriate primers. The Phobos output data as well as the designed primers were stored in the MySQL database. The respective tables were queried to output total numbers and coverage of tandem repeats and numbers of loci with potentially suitable primers.

### Searching for mtDNA

For the identification of mitochondrial DNA (mtDNA), all assembled contigs and single-read contigs of individual species were converted to a BLAST database (BLAST+ package version 2.2.25+, [34]). Mitochondrial genome sequences of closely related species deposited in GenBank were used as queries for local BLASTn and tBLASTx searches against this BLAST database.

Contigs in the database that had BLAST hits with an e-value ≤10^−12^ for a given query were assembled using Geneious version 5.4.6 [43]. The resulting contigs were inspected manually as described in [26]. Every scaffold was examined by BLAST searches against GenBank, and proteins and rRNAs annotated accordingly. tRNAs were annotated using tRNAscan-SE 1.21 [53] and ARWEN 1.2 [54].

### Searching for nuclear genes

To obtain functional information on nuclear-encoded proteins, we analysed our data (the contamination-free dataset: see below) with aid of the KEGG Automatic Annotation Server (KAAS [55]) (August 2012). We utilized the online version of KAAS with the single-directional best-hit method and default score thresholds. The results, i.e. the KEGG-Orthology assignments for individual contigs, were uploaded into our database and the hits were further classified according to the BRITE functional classification [56] retrieved via the public services provided by KEGG [57]. Each KEGG-Orthology record can potentially map to different BRITE-classes and this problem of inherent redundancy was resolved with a simple weighting system: each BRITE-class assigned to a contig was given a score equal to the number of reads for the contig divided by the number of pathways for that contig. BRITE-classes related to higher-level groups “Organismal Systems” and “Human Diseases” as well as the class “Enzyme Families” were ignored when creating the frequency charts, since the functional annotations were too imprecise for our data.

Furthermore, to obtain an independent estimate of the number of contigs with high similarities to known proteins, BLASTx searches against the Swiss-Prot database [58] were performed (October 5th, 2011) with a threshold e-value of ≤10^−12^. Only the best hits were collected and stored in the MySQL database. These data were used to obtain information about non-eukaryotic sequences and sequences derived from known mobile elements (interspersed repeats) (see below) and to produce “contamination-free” datasets. Functional mapping of the BLASTx hits was performed with the aid of the KEGG-database (Kyoto Encyclopedia of Genes and Genomes, [57]). The database was accessed with a PHP-written client as follows: A GI-number (NCBI's GenInfo Identifier) of a matched sequence was mapped to the KEGG gene identification number with the aid of interface functions (UniProt Mapping web-service) provided by the UniProt database [59]. Using the KEGG web-service, the KEGG gene identification number was assigned to its respective KEGG-Orthology identifier that was subsequently used to make functional annotations according to the BRITE pathways functional classification [56]. The annotation data were added to the same MySQL database that stored the BLAST hits. This database served as a source for final data analysis, comparison, and the creation of the tables and figures. Each gene could potentially map to different pathways and this problem of inherent redundancy was resolved with a simple weighting system: each pathway assigned to a contig was given a score equal to the number of reads for the contig divided by the number of pathways for that contig. Pathways related to higher-level groups “Organismal Systems” and “Human Diseases” were ignored for the remainder of this study.

### Searching for rRNA genes

rRNA genes in the contigs were identified by conducting BLASTn searches on local computers against the nr Database of NCBI and extracting the best 20 hits. Definition lines and taxon information for the hits were obtained as outlined above. rRNA genes were detected in the MySQL database with a searching query for NCBI records explicitly containing one or more of the terms “rRNA; 18S; 28S; 5S; 5,8S; 5.8S; 23S; 25S; 17S; ribosomal RNA; rDNA; SSU; LSU; internal transcribed spacer; ITS1; ITS2; external transcribed spacer” in their descriptions.

### Searching for transposable elements

Similar to the searches for rRNA genes, potential transposable elements in the contigs were identified by filtering the best BLASTn hits (case insensitive) for the terms “transposon, retrotransposon, transposable element, interspersed element, interspersed repeated mobile element, SINE sequence, SINE Alu, SINE family, LINE family, LINE sequence, Alu repeat”. The terms “transposon” and “retroposon” were searched for in the “species” name field. If one of the terms “flanking region”, “flank_region”, “flanking end” occurred in the definition line, the hit was excluded from consideration.

### Searching for contamination

#### Viruses

To account for possible viral contamination, BLASTx searches against the NCBI RefSeq Virus genomes Proteins Database were performed (viral*.protein.faa.gz, access date 09.09.2011). To avoid possible false positives (i.e. hits against loci similar to viral proteins, but not of viral origin) a very conservative approach with a maximum e-value of 10^−60^ was chosen.

In addition, we used the web version of the software DeconSeq [29] exploring the whole range of parameter combinations (coverage from 1× to 100×, identity from 60% to 100%). Both parameters were incremented by steps of one, resulting in 4,099 tested parameter combinations used to detect hits against viruses in the genomic library of *Metopaulias depressus* (data available on request).

#### Prokaryotic DNA

The data on prokaryotic contamination were obtained with the same BLASTn searches described in the “Searching for protein-coding nuclear genes” section. Taxonomic information was used to find sequences of prokaryotic origin. SQL and custom PHP scripts were utilized to obtain summary statistics concerning the numbers of reads and contigs assigned to respective groups, frequency charts coloured according to respective prokaryotic phyla and lists of highly frequent bacterial species. Life-history characterization of bacteria for a chosen library of *Austropallene cornigera* was performed manually through inspection of the relevant literature (see Supporting information S7).

**Figure 1:**
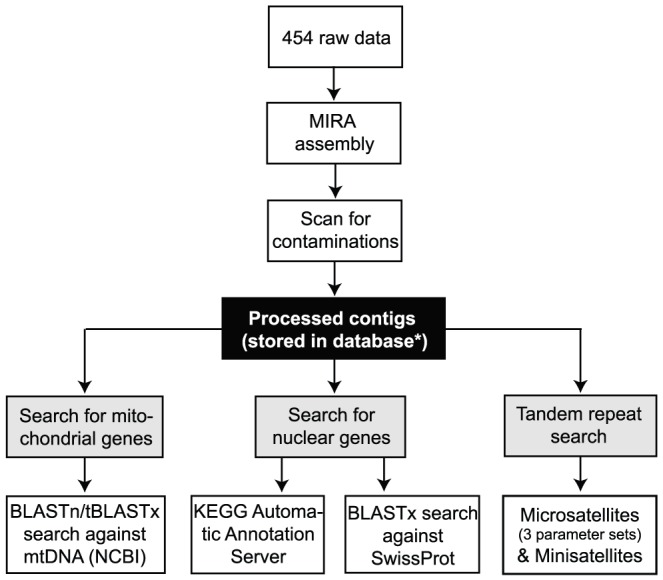
Workflow showing the methodological approach followed in this study. In this study we used a MySQL database (*) for storing the contigs. Other database formats are possible or reads can also be stored locally without a specific database.

An overview of the methodological workflow is presented in Figure 1. The data for this study can be viewed at http://www.evoeco.de.

## Results

### Sequencing statistics/assembly

Read number per genomic library ranged from 49,802 in Antarctic krill *Euphausia superba* to 339,640 in the vent limpet *Lepetodrilus* sp. nov. The total number of base pairs for the clipped reads ranged from 12,098,817 (*Euphausia superba*) to 105,577,603 (*Lepetodrilus* sp. nov., see Table 1). Average read lengths after quality clipping ranged from 211.5 bp (highly repetitive genome of the amphipod *Uristes adarei*) to 376.6 bp for the genomic library of the coral *Favia fragum*. In the microsatellite-enriched and length-selected libraries the average lengths were shorter (194.7 bp and 200.5 bp, for the bivalve *Lissarca notorcadensis* and the asteroid *Odontaster validus*, respectively). Even though approximately 5 µg of DNA were used consistently for library preparation, variation in read numbers obtained for the tagged libraries on the plates was high (Table 1) reflecting both the strong variation inherent in the technology (mainly library preparation) and differences in DNA quality.

Prior to producing the final assembly, we tested and compared different assembly settings and adjusted parameters for the MIRA assembler. To accommodate for the low-coverage situation we adjusted parameters and found that increasing the AL:mrs parameter to 85, while using the accurate *de novo* genome assemble mode of the MIRA assembler, produced high quality and conservative results. Increasing the AL:mrs stringency parameter reflected a trade-off between the low-coverage situation on the one hand and a known increased percentage of wrong base calls at read ends using a 454 sequencing approach and allelic variability on the other hand. In addition, the AS:mrpc parameter was set to 2, which means that at least two reads were needed to create a contig (see Material and Methods). The assembly resulted in a great number of assembled contigs, but most of the reads remained single-read contigs (Supporting information S2).

We estimated coverage ranges for the genomic libraries by using information on C-values from closely related organisms (deposited in the Animal Genome Size Database). Since in the case of our target species genome-size estimates were only available for one species, i.e. the Antarctic krill *Euphausia superba* [46], all other coverage approximations must be interpreted with caution. However, even when using the smallest genome size among related taxa for computing coverage values, we always found that only a small proportion of the genome has been sequenced. For the krill species *Euphausia superba*, our read library presents just a fraction of 0.03% of the genome (0.02% for contigs, see Table 2). Similarly low coverage values were estimated for the amphipod *Uristes adarei* based on a comparison to the uristid amphipod *Anonyx nugax* (genomic coverage in the library of only 0.06% for reads and 0.04% for contigs). For the species with most reads, the vent limpet, coverage estimates range from 5.5% to 20.1% for the reads (3.4% to 12.1% for contigs).

**Table 2:** Coverage estimations for the sequenced genomic libraries based upon genome size information of closely related taxa found in the Animal Genome Size database.

	C-values						Coverage estimations			
							for assembled data		for raw reads	
	Reference taxa	Values, pg					larger genome	smaller genome	larger genome	smaller genome
*Favia fragum*	Anthozoans	0.23	1.14				2.45%	12.12%	3.03%	15.00%
*Austropallene cornigera*	Pycnogonids	0.21	0.43	0.76			1.86%	6.74%	2.72%	9.84%
*Colossendeis megalonyx*	Pycnogonids	0.21	0.43	0.76			2.34%	8.46%	3.40%	12.30%
*Pallenopsis patagonica*	Pycnogonids	0.21	0.43	0.76			3.38%	12.24%	5.56%	20.14%
*Uristes adarei*	Uristidae: *Anonyx nugax*	27.00					0.04%		0.06%	
*Euphausia superba*	*Euphausia superba* *	48.50					0.02%		0.03%	
*Nematocarcinus lanceopes*	Caridea	range:	3.30	40.89			0.06%	0.78%	0.11%	1.33%
*Hyas araneus*	Majoidea	2.21	2.30	3.88	3.90	4.55	0.62%	1.29%	1.00%	2.06%
*Metopaulias depressus*	Sesarmidae	3.99	4.40				0.60%	0.66%	1.27%	1.40%
*Sericostoma personatum*	unknown						?		?	
*Lepetodrilus* sp.nov.	Lepetodrilidae	1.04	1.05	1.80			3.90%	6.75%	5.94%	10.29%
*Limatula hodgsoni*	Limidae: *Lima*	1.20	1.60				1.80%	2.40%	2.47%	3.29%
*Arctica islandica*	Close taxa: Veneridae+*Corbicula*	range:	0.96	2.30			0.78%	1.88%	0.91%	2.17%
*Gorgonocephalus chilensis*	Ophiuroids	2.20	2.30	2.40	3.00	3.30	0.45%	0.68%	0.57%	0.85%

* Information on genome size of *Euphausia superba* is based upon the flow-cytometry estimates listed in [46].

### Genetic markers detected

#### Tandem repeats

**Figure 2:**
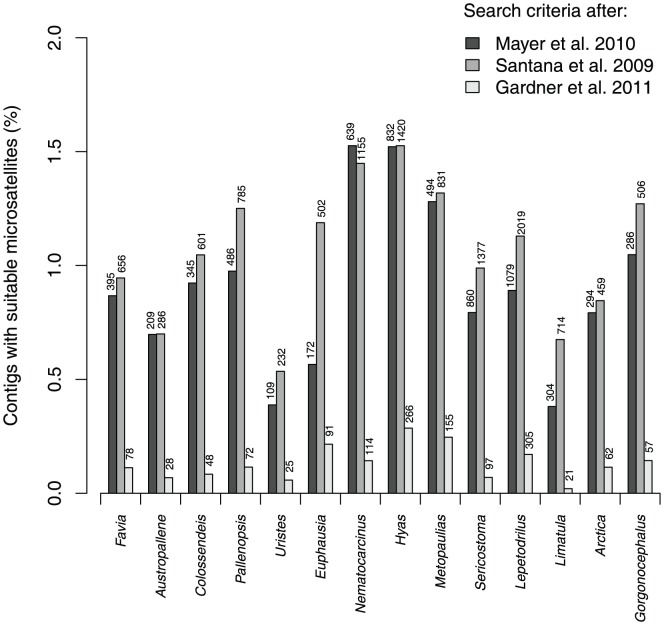
Percentage of contigs with candidate microsatellites found in the non-enriched libraries with three different search parameter settings. Search parameter settings were adapted from the three studies [4], [15], [48] and used in Phobos [47] runs. Numbers on top of the columns represent the total number of perfect microsatellites retained after restrictive filtering for quality criteria.

Non-enriched genomic libraries generally mirror the microsatellite distribution in the genome [17]. Hence, the analysis of a large proportion of non-enriched genomic reads allows estimation of the genomic density of these repeats. By analysing the density of microsatellites in the contigs (including single reads), using the search parameters of Mayer et al. [48], we estimated densities for the individual libraries ranging from 2,080 bp/Mbp, i.e., 0.21% of the genome in the bivalve *Limatula hodgsoni* to 161,435 bp/Mbp, i.e. 16.1% of the genome in the amphipod *Uristes adarei*. With the proportion of tandem repeats recovered from the genome of *Uristes adarei*, we document the highest genomic microsatellite density reported so far for a metazoan genome (see [60] for a heteropteran species with a high microsatellite density in the unit size range of 2–10 bp, but detected with less restrictive search parameters). The actual numbers of microsatellites identified per library ranged from 1,961 (*Austropallene cornigera*) to 26,700 (*Hyas araneus*, see Supporting information S3). When applying strict filtering criteria, i.e., accepting only microsatellites with 100% perfection from the imperfect search with Phobos, the number of candidate loci and their total number decreased (see Supporting information S3), ranging from 1,239 perfect microsatellites in *Austropallene cornigera* to 13,625 in *Hyas araneus*. After primer design with Primer3 the number of suitable loci decreased further. Considering only single read contigs (to avoid potential paralogous loci) and rejecting low complexity priming regions, the number of candidate loci ranged from 109 in *Uristes adarei* to 1,079 in *Lepetodrilus* sp. nov. (see Figure 2, Table 3). In the highly repetitive genome of the amphipod *Uristes adarei* most of the many microsatellites discovered initially lacked a second flanking region or primers contained low complexity regions and therefore most (98.73%) microsatellite loci were discarded from the initially 8,607 microsatellites resulting in the listed 109 (1.27%) candidate loci retained, when using the search parameters proposed in Mayer et al. [48]. For the settings suggested by Santana et al. [15], 232 microsatellites, and 25 for the extremely restrictive search parameters used by Abdelkrim et al. [13] and Gardner et al. [4].

**Table 3:** Total number and genomic density of microsatellites found in the libraries before and after applying stringent filtering criteria (best primers, single read contigs only, see Supporting information S3 for further information).

	Mayer et al. (2010)			Santana et al. (2009)			Gardner et al. (2011)		
Species	Total Number	Density (bp/Mbp)	After stringent filtering, with primers	Total Number	Density (bp/Mbp)	After stringent filtering, with primers	Total Number	Density (bp/Mbp)	After stringent filtering, with primers
*Favia fragum*	1,837	1,485	395	3,358	2,487	656	610	920	78
*Austropallene cornigera*	1,239	2,188	209	2,015	2,900	286	271	945	28
*Colossendeis megalonyx*	3,063	8,346	345	9,418	18,753	601	2,584	12,430	48
*Pallenopsis patagonica*	3,255	3,391	486	5,821	5,112	785	987	2,406	72
*Uristes adarei*	4,294	70,628	109	12,632	147,413	232	9,145	140,908	25
*Euphausia superba*	1,792	11,301	172	4,689	19,187	502	1,972	15,851	91
*Nematocarcinus lanceopes*	7,144	6,864	639	20,719	19,680	1,155	6,752	10,960	114
*Hyas araneus*	13,625	29,673	832	35,209	61,375	1,420	17,714	50,778	266
*Metopaulias depressus*	6,612	10,248	494	14,724	21,824	831	6,406	15,622	155
*Sericostoma personatum*	6,950	3,644	860	12,609	5,737	1,377	2,981	2,992	97
*Lepetodrilus* sp. nov.	11,994	5,569	1,079	30,609	12,378	2,019	13,693	8,768	305
*Limatula hodgsoni*	1,575	1,022	304	3,456	1,796	714	214	285	21
*Arctica islandica*	1,591	1,937	294	2,563	2,688	459	483	881	62
*Lissarca* (enriched)	27,768	76,580	464	59,565	138,709	806	37,181	114,629	640
*Odontaster* (enriched)	44,370	120,505	416	86,309	201,394	701	61,859	176,862	852
*Gorgonocephalus chilensis*	1,545	2,378	286	3,791	4,604	506	795	1,775	57

Results are given for three different sets of search parameters which correspond to search parameters in: Mayer et al. [48] but filtering for perfect microsatellites only, Santana et al. [15], Gardner et al. [4].

**Figure 3:**
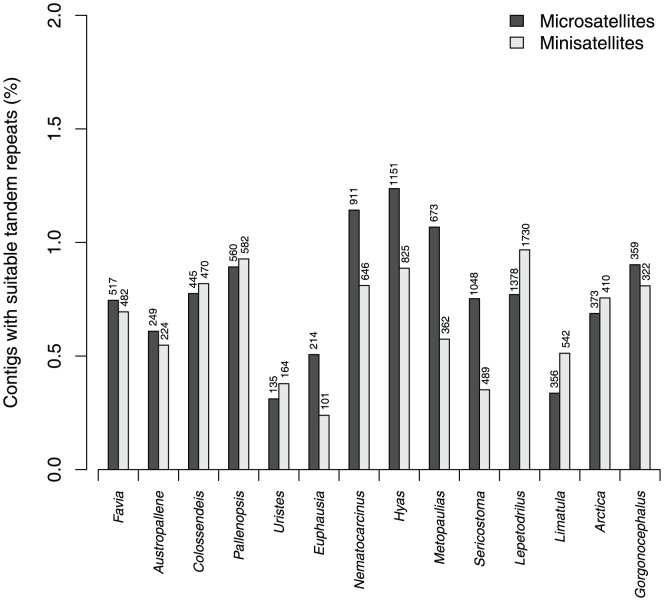
Percentage of contigs with microsatellites or minisatellites found in the non-enriched genomic libraries. Search parameters were according to Mayer et al. [48] used in Phobos [47]. For the analysis, repeats with a perfection greater or equal to 95% were retained. The numbers on top of the columns represent the total number found per library.

Minisatellites, i.e., repeats with a unit size of 7–50 bp, were found in all libraries (Figure 3, Supporting information S3). The coverage of minisatellites with a perfection of at least 95% ranged from 0.35% (3,529 bp/Mbp) in *Euphausia superba* to 10.34% (103,423 bp/Mbp) in *Colossendeis megalonyx* (see Supporting information S3). The number of minisatellites in single read contigs with appropriate flanking regions and primers ranged from 101 in *Euphausia superba* to 1,730 in *Lepetodrilus* sp. nov. For the enriched libraries, the number of microsatellites retained after strict filtering was in the range of the other libraries (64 for *Odontaster validus*, 4,347 for *Lissarca notorcadensis*). In two enriched libraries created for other taxa, the proportion of microsatellites was about 2 orders of magnitude higher, even after rigorous filtering (Supporting information S3).

### Mitochondrial DNA markers

**Figure 4:**
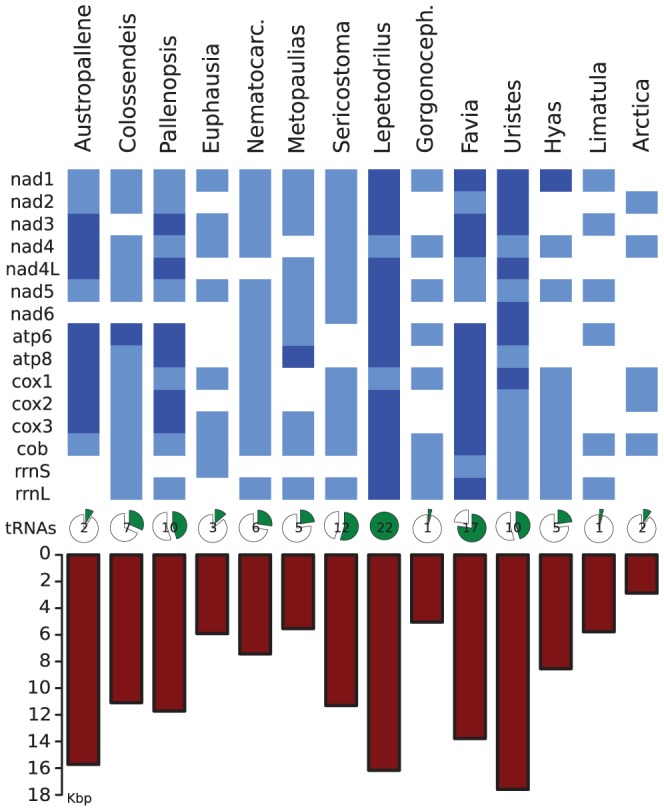
Overview over the different mitochondrial genes found in the non-enriched libraries. The upper section indicates full (dark blue) and partial (bright blue) mitochondrial protein-coding or rRNA genes recovered. The pie chart indicates the proportion and total number of tRNAs found. In the lower section the total contig lengths (in kb) of mitochondrial genes is shown.

**Figure 5:**
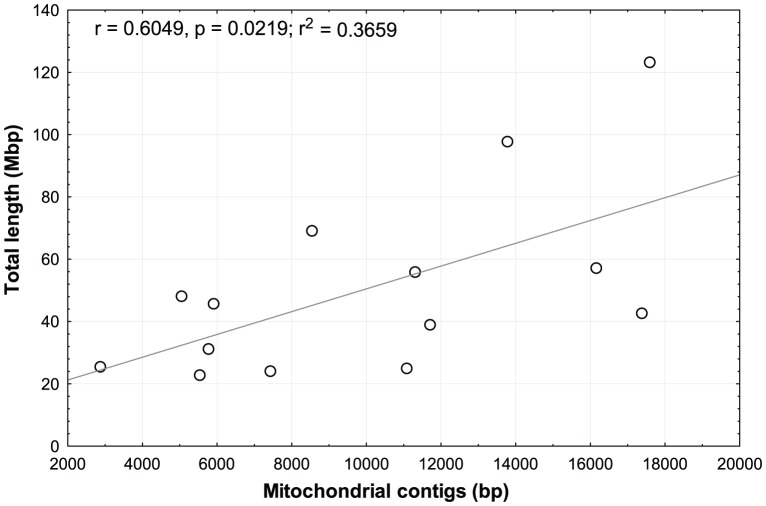
Correlation between genomic library size (y-axis) and total length of mitochondrial genome recovered (x-axis). A significantly positive linear correlation (Pearson r = 0.6049, P = 0.0219) between the number of base pairs sequenced and the proportion of the mitochondrial genome recovered was found.

All 14 genomic libraries contained mitochondrial DNA fragments (Figure 4). A significant positive correlation between the number of contig bp of the assembly and the number of mitochondrial bp found was detected (Spearman rank correlation: *r* = 0.6049, *P* = 0.0219, Figure 5). However, individual library success varied considerably and the number of recovered genes and tRNAs differed substantially. For the spider crab *Hyas araneus*, full or partial sequences of every mitochondrial gene including 22 tRNAs were found (Figure 4). In the microsatellite-enriched libraries not a single mitochondrial read was found as expected (see Supporting information S4).

From the 454 data complete or nearly complete mitochondrial genomes can be obtained by linking contigs via Sanger sequencing (see e.g. [26] for the *Colossendeis megalonyx* library).

Interestingly, in the case of the coral *Favia fragum* it was even possible to isolate not only the almost complete (15,718 bp) mitochondrial genome of the host but also a 1,663 bp fragment of the mitochondrial genome of its dinoflagellate symbiont *Symbiodinium* spp. (Supporting information S4).

The success of finding genes in a genetically uncharacterized taxon always depends on the availability and similarity of the data that are available for comparison. As an example, no mitochondrial hits were initially found for the genomic library of *Gorgonocephalus chilensis*. However, after the sequence of *Astrospartus mediterraneus* (GenBank Accession Number FN562580.1, [61]) was deposited in GenBank, 2,870 bp of mitochondrial contigs were found in the tBLASTx searches against the NCBI database and could therefore be classified as such.

### Nuclear DNA markers

Functional annotations performed with the KAAS pipeline allowed us to identify sequences with similarities to known proteins. The results showed that up to 2,772 contigs (for *Lepetodrilus* sp. nov.) had hits to known or predicted protein genes (Table 4). As expected, the microsatellite-enriched libraries (i.e. from *Lissarca* and *Odontaster*) showed the lowest percentage of identifiable protein-coding genes. Among the 14 genomic libraries the data obtained for the presumably more compact genomes (the coral *Favia fragum*, pycnogonids and molluscs (except for *Arctica islandica*)) showed higher values. A less sophisticated analysis utilizing BLASTx searches against the Swiss-Prot database showed comparable amounts of protein-coding sequences in our data, but overall lower than the values obtained with KAAS due to different candidate selection criteria.

**Table 4:** Summary of the nuclear gene identification in the genomic and enriched libraries.

Library	Type	Taxonomic Group	Total number of contigs	Number of contigs	
				With blastx hits against Swiss-Prot	With a KEGG Orthology number
*Favia fragum*	genomic	Cnidaria	69,405	1,529 (2.20%)	1,634 (2.35%)
*Austropallene cornigera*	genomic	Arthropoda	40,883	2,616 (6.40%)	1,717 (4.20%)
*Colossendeis megalonyx*	genomic	Arthropoda	57,425	1,273 (2.22%)	820 (1.43%)
*Pallenopsis patagonica*	genomic	Arthropoda	62,753	1,600 (2.55%)	1,057 (1.68%)
*Uristes adarei*	genomic	Arthropoda	43,336	370 (0.85%)	110 (0.25%)
*Euphausia superba*	genomic	Arthropoda	42,256	139 (0.33%)	127 (0.30%)
*Nematocarcinus lanceopes*	genomic	Arthropoda	79,740	737 (0.92%)	209 (0.26%)
*Hyas araneus*	genomic	Arthropoda	93,050	1,362 (1.46%)	566 (0.61%)
*Metopaulias depressus*	genomic	Arthropoda	63,040	2,789 (4.42%)	530 (0.84%)
*Sericostoma personatum*	genomic	Arthropoda	139,237	2,767 (1.99%)	1,639 (1.18%)
*Lepetodrilus sp. nov.*	genomic	Mollusca	178,788	2,868 (1.60%)	2,772 (1.55%)
*Limatula hodgsoni*	genomic	Mollusca	105,801	795 (0.75%)	756 (0.71%)
*Arctica islandica*	genomic	Mollusca	54,266	422 (0.78%)	446 (0.82%)
*Gorgonocephalus chilensis*	genomic	Echinodermata	39,809	462 (1.16%)	248 (0.62%)
*Lissarca notorcadensis*	enriched	Mollusca	84,498	45 (0.05%)	127 (0.15%)
*Odontaster validus*	enriched	Echinodermata	86,280	23 (0.03%)	34 (0.04%)

**Figure 6:**
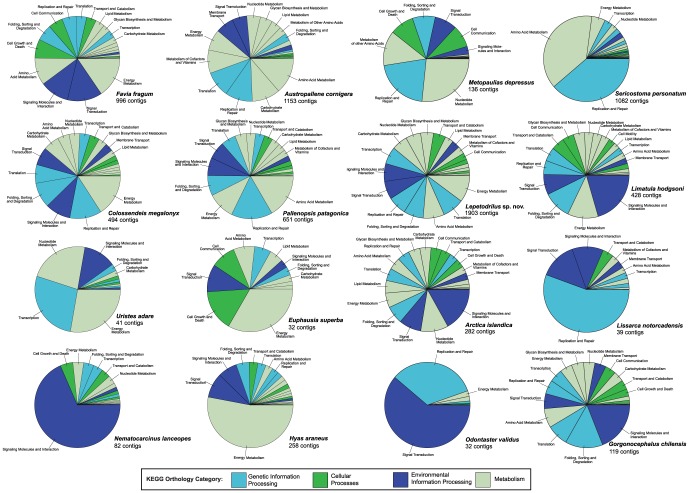
Hits of nuclear genes against KEGG BRITE Ontology database using the KAAS pipeline for the 16 genomic libraries. The number of hits is listed below the species name. Colours assigned according to the highest level of KEGG Orthology hierarchy (different organismal/cellular pathway groups/ecosystem processes).

The number and proportion of contigs that had tBLASTx hits to proteins in the Swiss-Prot database and the number of contigs with a K-number assigned by the KEGG Automated Annotation Server pipeline KAAS is given. A visual representation of the KEGG categories of the hits is given in Figure 6.

Functional classes identified by KAAS in our libraries are very diverse (Figure 6). For the genomes with large predicted sizes, in particular *Euphausia superba* and *Uristes adarei*, few hits to known protein-coding genes were found. For the other genomes, up to 1,903 hits to genes from the KEGG categories “Genetic Information Processing”, “Cellular Processes”, “Environmental Information Processing” and “Metabolism” were obtained. This information could be important for a wide range of molecular studies.

#### Ribosomal RNA genes

Ribosomal RNA (rRNA) genes were detected with the aid of BLASTn searches against GenBank and various rRNA genes were identified in the genomic libraries (Table 5). The number of positive rRNA gene hits ranged from 62 for *Uristes adarei* to 2,027 sequences for *Limatula hodgsoni*, adding up to a total contig length of 2,453 bp for *Uristes adarei* and 58,588 for the 633 sequences detected for *Gorgonocephalus chilensis*.

**Table 5:** rRNA genes found in the different libraries.

Library	Type	Taxonomic Group	Total contig length, bp	Number of reads	Avg reads number per contig	Number of contigs
*Favia fragum*	genomic	Cnidaria	17,606	700	31.82	22
*Austropallene cornigera*	genomic	Arthropoda	14,290	151	6.04	25
*Colossendeis megalonyx*	genomic	Arthropoda	25,166	506	23.00	22
*Pallenopsis patagonica*	genomic	Arthropoda	28,756	787	29.15	27
*Uristes adarei*	genomic	Arthropoda	9,314	62	3.26	19
*Euphausia superba*	genomic	Arthropoda	20,753	181	4.89	37
*Nematocarcinus lanceopes*	genomic	Arthropoda	21,651	1380	47.59	29
*Hyas araneus*	genomic	Arthropoda	36,624	827	20.17	41
*Metopaulias depressus*	genomic	Arthropoda	12,045	124	7.29	17
*Sericostoma personatum*	genomic	Arthropoda	55,832	637	8.27	77
*Lepetodrilus* sp. nov.	genomic	Mollusca	11,628	186	5.81	32
*Limatula hodgsoni*	genomic	Mollusca	42,545	2027	47.14	43
*Arctica islandica*	genomic	Mollusca	12,486	97	3.59	27
*Gorgonocephalus chilensis*	genomic	Echinodermata	58,588	633	10.91	58
*Lissarca notorcadensis*	enriched	Mollusca	2,453	96	7.38	13
*Odontaster validus*	enriched	Echinodermata	12,299	246	6.83	36

The total number of reads, the number of assembled contigs with coverage and the total unique rRNA gene bp are listed.

#### Transposable elements

In the libraries of the three pycnogonids and the two decapod species *Metopaulias depressus* and *Hyas araneus* we found 5–81 reads (1–9 contigs) with matches to known transposable elements (Table 6). In the genomic library of *Sericostoma personatum*, however, we found 1,895 reads (assembled to 243 contigs) with high similarity to *insect mariner* retrotransposons. This reflects a proportion of 0.75% of the reads. However, all of the species analysed in this study have a great phylogenetic distance from classical genetic model species with well-annotated transposable elements (data not shown). All more closely related species are only poorly, if at all, genetically characterized. Therefore, it is very likely that a major proportion of transposable elements in our genomic libraries went unnoticed.

**Table 6:** Characteristics of contigs with homology to known transposable elements in the libraries.

Library	Type	Taxonomic Group	Total contig length (bp)	Number of reads	Avg read number per contig	Number of contigs	Total read length (bp)	Main hits
*Favia fragum*	genomic	Cnidaria	0	0	0	0	0	
*Austropallene cornigera*	genomic	Arthropoda	5,955	81	10.1	8	20,220	*Rana (frog) Tc1*
*Colossendeis megalonyx*	genomic	Arthropoda	978	5	5.0	1	1,389	*Xiphophorus (fish) Rex3-retrotransposons*
*Pallenopsis patagonica*	genomic	Arthropoda	4,875	26	2.9	9	9,994	*Rana* (frog) Tc1; *Lepeophtheirus (copepod) Tc3*
*Uristes adarei*	genomic	Arthropoda	0	0	0	0	0	
*Euphausia superba*	genomic	Arthropoda	0	0	0	0	0	
*Nematocarcinus lanceopes*	genomic	Arthropoda	0	0	0	0	0	
*Hyas araneus*	genomic	Arthropoda	2,707	33	16.5	2	11,246	Galatheid (decapods) GalEa transposon
*Metopaulias depressus*	genomic	Arthropoda	1,765	7	2.3	3	3,291	*Litopenaeus (decapod) non-LTR retrotransposon I-type; insect Mariner-transposons*
*Sericostoma personatum*	genomic	Arthropoda	250,720	1,895	7.8	243	642,767	insect Mariner-transposons
*Lepetodrilus* sp.nov.	genomic	Mollusca	0	0	0	0	0	
*Limatula hodgsoni*	genomic	Mollusca	0	0	0	0	0	
*Arctica islandica*	genomic	Mollusca	0	0	0	0	0	
*Gorgonocephalus chilensis*	genomic	Echinodermata	0	0	0	0	0	
*Lissarca notorcadensis*	enriched	Mollusca	0	0	0	0	0	
*Odontaster validus*	enriched	Echinodermata	0	0	0	0	0	

### Non-target organism DNA

We systematically searched for traces of DNA not belonging to the organism under study. In particular, we searched for expected symbionts and for bacterial and viral contamination. For each section we will here highlight cases in which the contamination was particularly prominent.

#### Symbionts

From the coral *Favia fragum,* tissue was extracted that contained a DNA mixture of the host *Favia fragum* and its symbionts belonging to the dinoflagellate genus *Symbiodinium*. Therefore, the DNA could potentially include DNA of the nuclear and mitochondrial host genome, the nuclear and mitochondrial symbiont genome, as well as the plastid genome of the symbiont. The results of the mitochondrial DNA marker detection revealed 15,718 assembled bp of mitochondrial reads for the coral and 1,663 bp for the symbiont (Supporting information S4).

To explicitly distinguish between nuclear and plastid DNA of host and symbiont we performed BLASTn searches of all “Faviinae” and “Dinoflagellata” sequences as well as the newly sequenced genome of the cnidarian *Nematostella vectensis* obtained from GenBank (access date 24.09.2011; for exact search terms see Supporting information S5) against the 77,440 *F. fragum* tissue contigs (42,696,657 bp) as a database. We counted and assigned the hits with an e-value not exceeding 10^−12^ (Supporting information S5) resulting in 434 contigs with at least one hit. Of all contigs 17 had matches to more than one of the genomes of interest. All these cases indicate erroneous annotations in the database. In addition, ten contigs had only hits against plastid sequences, 14 contigs had exclusive hits against the dinoflagellate genome and 393 contigs had hits against coral DNA only. Together with the results from the mitochondrial DNA these findings indicate that even low-coverage genome surveys may allow the identification of phylogenetically different genomes hidden within one organism.

#### Bacteria

**Figure 7:**
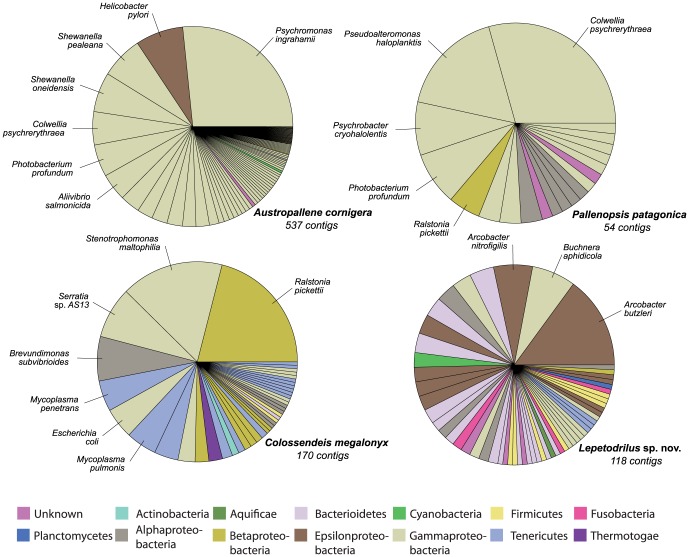
Bacterial hits found in four genomic libraries. Hits for the bacterial species are displayed next to the chart pie for species with ≥4% of the hits. For the three pycnogonid species, Gammaproteobacteria are predominant, whereas for the vent limpet *Lepetodrilus* sp. nov. different bacterial groups were detected. The colours of the charts relate to the phyla/classes of Bacteria (see legend).

Up to 1.57% of the reads (1.31% of the contigs) in the libraries had highest similarity to bacterial DNA. Most hits were found for the three analysed pycnogonid species *Austropallene cornigera* (*n* = 537), *Colossendeis megalonyx* (*n* = 170) and *Pallenopsis patagonica* (*n* = 54), but bacterial DNA was also recorded in the vent limpet (*n* = 118, see Figure 7). Analysing the bacterial hits for the pycnogonid libraries showed that most had closest matches to various Gammaproteobacteria, whereas for the vent limpet the bacterial origin was very diverse (Figure 7, Supporting information S6). The diversity of bacterial species reported by the searches was high. For *Austropallene cornigera*, an Antarctic species, most of the hits were assigned to strains of *Psychromonas ingrahamii*, a cold-adapted species known from Arctic waters (Supporting information S7). Furthermore, our data revealed many reads with hits to various species of *Shewanella*, which are predominantly found in deep-sea habitats. Interestingly, 89 reads were assembled to one contig that had the best match with *Helicobacter pylori,* a species commonly known from human stomachs where it induces gastritis [62]. Other abundant bacteria were also free-living, commensalic or pathogen bacteria that have been reported from various marine, often either deep-sea and/or cold-water environments.

#### Viruses

BLASTx searches against NCBI RefSeq Virus genomes Proteins Database yielded hits in five libraries (Table 7). Most exceptional in terms of number of hits against virus sequences was the bromeliad crab *Metopaulias depressus.* Here, we identified 14,131 reads (7.56% of total reads) with hits to the White-Spot-Syndrome-Virus (WSSV) that is well known primarily from penaeid shrimp aquaculture and repeatedly reported for other decapods and even for other crustacean groups [63], [64]. For a more accurate assignment, we took the WSSV genome (gi: 17016399) as a query and performed tBLASTx searches with an e-value threshold of 10^−12^ against all assembled and one-read contigs in the genomic libraries of *M. depressus*. The tBLASTx approach revealed that 9.23% of the sequenced DNA had hits and thus resemble WSSV-related viruses. Interestingly, the already sequenced WSSV consists of only 292,967 bp in 531 ORFs, whereas we have found 453,318 unique bp in this study. It cannot be excluded that horizontal gene transfer has contributed to the pattern observed in *Metopaulias depressus*.

**Table 7:** Characteristics of contigs with homology to viral protein sequences in the libraries.

Library	Type	Taxonomic group	Total contig length (bp)	Number of reads	Avg read number per contig	Number of contigs	Total read length (bp)	Best hits
*Favia fragum*	genomic	Cnidaria	0	0	0	0	0	
*Austropallene cornigera*	genomic	Arthropoda	0	0	0	0	0	
*Colossendeis megalonyx*	genomic	Arthropoda	449	3	3.0	1	1,073	Enterobacteria phage lambda (tail component)
*Pallenopsis patagonica*	genomic	Arthropoda	0	0	0	0	0	
*Uristes adarei*	genomic	Arthropoda	0	0	0	0	0	
*Euphausia superba*	genomic	Arthropoda	0	0	0	0	0	
*Nematocarcinus lanceopes*	genomic	Arthropoda	0	0	0	0	0	
*Hyas araneus*	genomic	Arthropoda	1,927	19	19.0	1	7,747	*Cotesia congregata* bracovirus (hypothetical protein)
*Metopaulias depressus*	genomic	Arthropoda	447,712	14,131	147.2	96	5,253,117	**White spot syndrome virus**
*Sericostoma personatum*	genomic	Arthropoda	8,674	145	72.5	2	48,174	Strawberry vein banding virus (retrotransposase), *Vaccinia* virus (ribonucleotide reductase)
*Lepetodrilus* sp.nov.	genomic	Mollusca	468	1	1.0	1	468	*Emiliania huxleyi* virus 86 (ribonucleoside-diphosphate reductase)
*Limatula hodgsoni*	genomic	Mollusca	0	0	0	0	0	
*Arctica islandica*	genomic	Mollusca	0	0	0	0	0	
*Gorgonocephalus chilensis*	genomic	Echinodermata	0	0	0	0	0	
*Lissarca notorcadensis*	enriched	Mollusca	0	0	0	0	0	
*Odontaster validus*	enriched	Echinodermata	0	0	0	0	0	

Using the DeconSeq software with different parameter combinations, only between 0.51% (n = 322) and 5.95% (n = 3749) of the proposed virus contaminant reads were found that had initially been detected by BLAST searches. All hits found with standard settings (coverage 90×, identity ≥94%) belonged to repetitive regions or even consisted solely of a tandem repeat. No sequence of the WSSV-related virus was detected with default settings. In the least restrictive search (coverage 1×, identity ≥60%), only 30 of the 3,749 DeconSeq hits were contigs identified using the BLAST approach. All others seem to be false positives (mainly tandem repeats).

## Discussion

For all 14 genomic libraries analysed, the sequence coverage was just a minor fraction of the total genome. Estimated coverage values ranged from 0.1 to 20%. Our results highlight the great potential of such low-coverage next-generation sequencing data for the simultaneous analysis of multiple genetic markers supplementing primary results of Rasmussen and Noor [25]. Moreover, for the first time we systematically compare the impact that different non-target DNA sources may have on analysed libraries. The approach we advocate differs in one fundamental aspect from most other studies (e.g., [4], [14], [15], [25]): prior to the main analyses of the low-coverage data, an assembly was performed to reduce redundancy. Although for average coverage values of <1 it may seem unlikely that overlapping reads exist, it turns out that several genomic fragments are highly overrepresented and form rather long contigs. We found this to be relevant for rRNA genes (Table 5), mitochondrial genes (Figure 4, Supporting information S4), transposable elements (see for examples the mariner retrotransposons, Table 6) but also for other, possibly single-copy nuclear genes (Table 4). Hence, the strategy of using a stringent assembly with repeats masked to avoid merging reads that are not from the same physical locus is important to prepare the data for all subsequent steps. In a few cases (1–4% of the contigs) MIRA did not mask terminal repeats, leading to some potentially erroneously assembled contigs. Attempts to assemble the data without masking internal repeats using the assembler available in the commercial software Geneious led to artifactual results, since several reads ending with the same tandem repeat were assembled. We therefore suggest to assemble the reads only with a software capable of masking repeats prior to the assembly process to prevent unlinked contigs being joined artificially by paralogous repeat regions.

For our study we used 454 pyrosequencing as the sequencing technique. This provides comparatively fewer but longer sequences as compared to most other high-throughput technologies [65], in particular when comparing it to Illumina sequencers. The advantage of Illumina is that a much greater coverage can be obtained. The short reads have the drawback that microsatellite development is more difficult and homology searches are less informative. It has been demonstrated that the disadvantage of short reads can be compensated effectively by using paired-end Illumina sequences [66].

### Tandem repeats

Different studies have used different search criteria for defining microsatellites (see [48] for discussion). Hence, the computed tandem repeat contents are difficult to compare. In this study we used three different published sets of search parameters to detect microsatellites [13], [15], [48] and compared the results. Whereas most repeats were reported for the parameters used by Santana et al. [15], a much lower number was found when applying the rather restrictive criteria used by Abdelkrim et al. [13] and Gardner et al. [4].

Strict filtering criteria led to a decrease in obtained microsatellites mainly due to short read/contig lengths, which in turn led to absent flanking regions (see also [13], [14], [67], [68]). These strict filtering criteria led to a significant dropout of candidate loci for the three different search parameter settings (see Supporting information S3). The extremely strong decrease in the candidate microsatellites with suitable primers found in the Southern Ocean amphipod *Uristes adarei* (only 1.95% of the initially found candidates retained) was mainly due to microsatellites in this highly repetitive genome, that were lacking a second flanking regions because of great repeat length.

Although the choice of appropriate search parameters still remains a subject of controversial discussion, it is obvious that for all search parameter sets, even with very stringent filtering criteria (i.e. perfect microsatellites filtered from an imperfect Phobos search, considering only single reads with appropriate flanking regions) the total number of reads containing suitable candidate loci was sufficient for many candidate microsatellites ranging from 109 (2.53% of the microsatellite candidates) in *Uristes adarei* to 1,085 (8.98%) in the vent limpet *Lepetodrilus* sp. nov. In general, for molecular ecological or population genetic studies on non-model organisms, microsatellites have usually been obtained by enriching genomic libraries, cloning and shotgun Sanger sequencing of these fragments [69], [70], [40]. However, due to recurrent PCR amplifications, the redundancy is often considerable and the number of clones that can be sequenced is limited due to the involved costs (about 5 USD per plasmid prep and sequence read). For high-throughput sequencing data, the cloning step can be avoided and sequencing costs have decreased to less than 0.02 USD per 454 read, with an average clipped length of 272.02 bp in our examples. With increasing throughput and sequence lengths, next-generation sequencing platforms such as 454, Illumina or the Ion-Torrent, facilitate marker development drastically. In particular, when applying the strict filtering criteria and when scanning for problematic reads, the polished high-throughput sequencing datasets are superior to classical approaches. An additional benefit of using this methodology is that microsatellites in the vicinity of coding genes (in particular within 5′ and 3′ UTRs) can be designed and compared to putatively neutrally evolving microsatellites in intergenic regions. Microsatellites in coding regions (i.e. non-neutral markers) reflect the selection regime prevalent in populations/species and can be used to identify functional traits that explain evolutionary differences.

The sequenced libraries that were enriched for microsatellites yielded many more microsatellite loci than the non-enriched genomic libraries. However, all our non-enriched libraries provided sufficient unique and suitable microsatellite loci to work with in subsequent studies (46 unique contigs with suitable microsatellites for the genome of *Uristes adarei*), even with extremely restrictive filter settings. This is in agreement with former comparisons of enriched vs. non-enriched 454 libraries in two case studies of non-model and model organisms ([68] for *Acacia harpophylla*, [16] for *Apis mellifera*).

### Mitochondrial genes

For all species, several mitochondrial gene fragments were identified, although the overall yield differed considerably. For the individual taxa, between 2,870 bp and 16,158 bp of the mitochondrial genomes were recovered. These differences may be due to the extraction of different tissue types (Supporting information S1), since the copy number of mitochondrial DNA per cell can vary among different tissues [71]. Furthermore, difference in the nuclear genome size may also impact the proportion of recovered mitochondrial genome fragments. For the Antarctic krill species *Euphausia superba,* Jeffery (2010) documented an abnormally large genome size [46]. For this species, a particularly low proportion of mitochondrial gene fragments was recovered (5,502 bp) which might be a consequence of dilution effect due to large nuclear genome size. In other studies on invertebrates, comparable or slightly higher proportions of mitochondrial DNA were recovered from 454 libraries [25], [72]. Completing the mitochondrial genomes by Sanger sequencing on the basis of sequences obtained in this study was trivial for *Colossendeis megalonyx*, *Sericostoma personatum*, *Austropallene cornigera,* and *Pallenopsis patagonica* [26]. Compared to primer walking approaches with often unpredictable outcomes (see discussion in [24]), we instead suggest to invest in high-throughput sequencing as demonstrated in this study or by Groenenberg et al. [73].

### Non-target genome DNA

Even though low-coverage genomic surveys represent only a minor fraction of the genome, they offer a great potential for evolutionary biologists. Solely extracting markers in a traditional way, i.e. picking those that look appropriate without doing a sophisticated analysis of the whole large dataset, may result in overlooking interesting and important phenomena, such as DNA of other organisms (viruses, bacteria, symbionts). Furthermore, primers may be designed for microsatellites located in mobile DNA elements in the genome, which leads to genotyping problems. We have demonstrated that with some effort, these important elements can be identified in order to maximise the use of the polished high-throughput libraries.

In the process of developing genetic markers it is commonly assumed that the presence of non-target DNA is negligible and hence requires no sophisticated action. However, contamination is a severe problem in genetic research [32], [74] and many different sources of contamination of the target DNA exist. In this study we used a BLAST approach to quantify the (minimum) amount of non-target DNA in the analysed libraries. Other bioinformatic approaches to detect contamination had been tested in phylogenomic studies and were found to be superior to BLAST in terms of speed [29]. In particular, approaches that align short reads against a known reference sequence of the potential contaminants using Burrows-Wheeler Transform (BWA) are described as powerful [36]. Using the software DeconSeq [29], which utilizes the BWA, we could only detect a small subset of the virus contaminant in our library of *Metopaulias depressus,* but found a huge number of false positive, repetitive hits. In contrast, the BLAST approach identified 9.23% of the total number of reads as originating from WSSV-related viruses and thus should be classified as a contamination. The comparatively low success of DeconSeq seems to be due to the low similarity of the virus found in *Metopaulias depressus* and the WSSV reference genome. In exploratory studies on non-model organisms from weakly characterized habitats, as in our study, reference genomes for potential contaminants do not exist. Therefore, slower but more thorough approaches such as a combination of different BLAST searches, as outlined above, can be superior over DeconSeq. Although slower, BLAST was able to identify the WSSV-related virus in the *Metopaulias depressus*. Our datasets may serve as a source for further benchmark tests, similar to the study of Schmieder and Edwards [29].

Evidence for the presence of symbionts was obtained for the hard coral *Favia fragum.* Here, the tissue extracted from organism can potentially contain five genomes 1) the nuclear genome of the coral host *Favia fragum*, 2) the mitochondrial genome of the coral host, 3) the nuclear genome of the symbiont *Symbiodinium* sp., 4) the mitochondrial genome of the symbiont, and 5) the chloroplast genome of the symbiont. This complex mixture of genomes is usually avoided in coral studies. Researchers use larval tissue (e.g. [75]) or sperm (e.g. [76]) to enrich the amount of host DNA and minimize the presence of symbiont and mitochondrial genomes. Our study, however, found that including the holobiont might provide a lot of additional data without necessarily reducing the level of information obtained from the target host species. Even without enriching the extracted tissue for the host nuclear DNA, the majority of obtained reads/contigs belonged to the host genome (*Favia fragum*). In addition, a very large proportion of the *Favia* mitochondrial genome could be assembled providing valuable additional markers as well as a very good basis for mitogenome completion using conventional Sanger sequencing. For the symbiont mitogenome, an important mitochondrial marker (CO1) could be identified. Summarizing, we can conclude that the presence of several different genomes enhances the amount of information that can be obtained from low-coverage genome surveys.

With respect to viral reads, we found several instances in which the amount of non-target DNA was considerably high with a contribution of up to 10% of the total number of sequenced base pairs in the library (as in *Metopaulias depressus*). Viruses are capable of infecting organisms from all evolutionary lineages and actually do so very frequently [77]. Hence, genomic traces of viruses, the “virome”, have been reported from genomic libraries, particularly from sequenced model organisms and revealed a huge diversity (e.g. [78]).

We found that bacterial reads were present in a non-negligible proportion in four of our libraries. Interestingly, three of these libraries were from pycnogonids and one from a hydrothermal vent limpet. Pycnogonids have a special anatomy in that their organs are shifted mainly into their legs, due to their very small trunk. This, however, enhances the risk of including gut content within the extracted DNA. Although we used only the tissue from the distal leg parts in Co*lossendeis,* we had to grind whole legs for *Austropallene* and *Pallenopsis* to achieve the necessary amount of DNA. Bacterial contamination, in particular in the latter two pycnogonids, very likely stems from ingested marine bacteria. Reads identified as bacterial contamination in the three pycnogonids usually had the closest matches to Gammaproteobacteria, which are cold-water adapted prokaryotes. Although studies of the molecular diversity of bacterial communities in the Southern Ocean are in their infancy [79], preliminary data show that the bacterial species differ from those in other oceans and have typical adaptations to the constantly cold marine environment. Species found in pelagic bacterial culture collections from the Southern Ocean frequently belong to Gamma- or Alphaproteobacteria [79]. This view is mostly consistent with the hits observed in our library. Interestingly, the number of different bacteria we found was high and only few redundant reads were found, further highlighting the enormous bacterial diversity. One highly redundant contig, composed of 89 single reads, had the best match against the gram-negative bacterium *Helicobacter pylori*. Although *Helicobacter pylori* is not only known from human intestines, but from different aquatic habitats including marine habitats [80], the strong overrepresentation of one fragment suggests that it may result from a contamination of the library. The limpet *Lepetodrilus* sp. nov. grazes on bacterial films in the vent habitat. Thus contamination by bacteria attached to the tissue processed is a likely explanation. Rogers et al. [81] investigated the bacterial communities in the vent habitat by 16S rDNA clone library sequencing. They found a high proportion of Gammaproteobaceria, Alphaproteobacteria, Bacteroidetes and Deferribacterales. With the exception of the latter, these groups were also represented in our identified hits. In addition, Epsilonproteobacteria, in particular bacteria of the genus *Arcobacter*, were found several times.

In principle, lateral gene transfer between symbiotic bacteria and eukaryotic genomes could be a further explanation for the data, since it may be more common than expected and may even be of functional importance in the course of evolution [82]. A major argument against this possible explanation is the fact that most of the closest hits in the bacteria were species that are free-living. Although several libraries were not obviously affected by bacterial reads, the contribution of 1.57% in the library of *Austropallene* advises caution and highlights the importance of testing for contamination prior to subsequent analyses.

Cases of contamination by other eukaryotic species were rare, but present (e.g. the presence of a dragonfly sequence, although this template was not extracted from any of the authors' laboratories). Clearly, such an unexpected contamination needs to be taken into account by active searching. This issue is further complicated for eukaryotic symbionts. Here, successfully finding a certain non-target DNA depends on a homolog sequence being deposited in the database that is used for contamination screening. Consequently, an unknown proportion of the libraries may originate from so called “dark matter” sequences of other species that are not represented in the public databases.

## Conclusions

Using examples from 14 low-coverage genomic 454 libraries, genetic markers for population genetic analyses as well as for phylogenetic studies or other biological disciplines were identified and characterised. We suggest a series of steps which are critical to avoid some of the problematic pitfalls of processing low coverage libraries for evolutionary biology. We recommend an initial stringent assembly of the reads as a key step for reducing redundancy and increasing per locus information content, even in low coverage surveys. Masking repeats prior to assembly is important to avoid merging unrelated reads that are united by similar repeat motifs. Prior to downstream analyses of sequence data, it is important to validate the origin and identity of sequences. Although for uncharacterized genomes little information on sequence identity is available in public databases, we have demonstrated that a significant proportion of library reads were of non-target origin, using simple BLAST routines. If not excluded from the libraries prior to downstream analyses, such contaminant reads can lead to biased or even strongly misleading inferences of evolutionary processes from the contaminated data.

## Supporting Information

Supporting information S1 **Information on sampling sites, tissue and DNA extraction protocols for the specimens analysed in this study.**
(PDF)

Supporting information S2 **Overview over the assembly results for the different genomic libraries.** The number of contigs (y-axis, log-scale) with the respective number of reads included in the contig (x-axis). In all cases, single-read contigs (x = 1) represented the majority of contigs after assembly.
(PDF)

Supporting information S3 **Information on the microsatellites found (total number, bp, density, filtered candidate loci with primers).** For the 95% perfection analyses we searched for imprefect microsatellites/minisatellites and filtered out only those with a perfection equal or higher than 95%.
(XLS)

Supporting information S4 **Overview over the different mitochondrial genes found in the different libraries.** An ‘f’ indicates that the whole gene was found whereas ‘p’ indicates that only a part of the gene was found.
(XLS)

Supporting information S5 **Analysis of the contigs from the** ***Favia fragum*** **454 library.** The number of BLASTn hits against either dinoflagellate (GenBank Taxon ID “Dinoflagellate” NOT gene_in_plastid_chloroplast[PROP]), dinoflagellate plastome (GenBank Taxon ID “Dinoflagellate” AND gene_in_plastid_chloroplast[PROP]) or coral (GenBank Taxon ID “Faviinae” and the *Nematostella vectensis* genome). The first 6 contigs had hits for both, nuclear and plastid dinoflagellate fragments. 11 contigs had hits for nuclear dinoflagellate and coral fragments. For information on the mitochondrial genes found for the coral and the symbiont see Supporting information S4.
(XLS)

Supporting information S6 **Taxonomic classification of bacterial hits found within the three pycnogonids and the vent limpet** ***Lepetodrilus*** **sp. nov. (see also** **Figure 7** **).**
(PDF)

Supporting information S7 **Bacterial hits for** ***Austropallene cornigera*** **obtained with BLAST and respective information on habitats.** References are found in the second worksheet (“References”).
(XLS)
