# Supplementary material for: Exploring Pandora's Box: Potential and Pitfalls of Low Coverage Genome Surveys for Evolutionary Biology
Source: PLoS One. 2012 Nov 21;7(11):e49202. doi: 10.1371/journal.pone.0049202 (PMC3504011; doi:10.1371/journal.pone.0049202)
Supplement: Supporting information S1 — Information on sampling sites, tissue and DNA extraction protocols for the specimens analysed in this study. (PDF) [file pone.0049202.s001.pdf]

Supporting information 1: Information on sampling sites, tissue and DNA extraction protocols for the specimens analysed in this study.

Libary	Description of extraction protocol
Favia fragum	Originally, the Favia individual came from Curaçao. Tissue was scraped off an area of 3 by 3 mm and DNA was extracted using the Qiagen DNeasy Blood and Tissue kit following the providers instructions. DNA integrity was confirmed on a 1% agarose gel and the concentration was determined using an Implen photometric cell.
Austropallene cornigera	Specimens were sampled during the ICEFISH 2004 expedtion from Bouvet Island. Sampling details: Station 59 Otter Trawl 44, 26.06.2004. Tissue was extracted from Tibia1, Tibia2, Tarsus, Propodus of 4 walking legs using Qiagen the DNA Blood & Tissue kit according to the manufacturer's protocol.Integrity of DNA was verified on a 1.5% TBE agarose gel and concentration determined on a Implen photometric cell.
Colossendeis megalonyx	Specimen was sampled during the ICEFISH 2004 expedition from Bouvet Island waters. Sampling details: Station 76, Otter Trawl 50, 28.06.2004. Tissue was extracted from Tibia1, Tibia2, Tarsus, Propodus of 2 walking legs using Qiagen the DNA Blood & Tissue kit according to the manufacturer's protocol. Integrity of DNA was verified on a 1.5% TBE agarose gel and concentration determined on a Implen photometric cell.
Pallenopsis patagonica	Specimen was sampled during the ICEFISH 2004 expedition from the Falkland Islands (Ppa E010). Tissue was extracted from Tibia1, Tibia2, Tarsus, Propodus of 4 walking legs using Qiagen DNA Blood & Tissue kit according to the manufacturer's protocol. Only 100 ul AE buffer was used for elution. Integrity of DNA was verified on a 1.5% TBE agarose gel and concentration determined on a Implen photometric cell.
Uristes adarei	CEMARC expedtion (2008-09) to Terre Adelie. Station 1472. Tissue was extracted from all walking legs and 2 pleopod I using Qiagen DNA Blood & Tissue kit according to the manufacturer's protocol. Only 100 ul AE buffer was used for elution. Integrity of DNA was verified on a 1.5% TBE agarose gel and concentration determined on a Implen photometric cell.
Euphausia superba	CEMARC expedition (2008-09). Date: 15.1.08 , Location: Terre Adelie (-66.166858, 139.6211), Antarctic<br>Tissue was extracted from legs using the Qiagen DNA Blood and Tissue kit according to manufacturer's protocol. Quality and quantity of DNA was checked on a Nanodrop (Thermo Scientific).
Nematocarcinus lanceopes	Tissue was extracted from legs using the Qiagen DNA Blood and Tissue kit according to manufacturer's protocol. Quality and quantity of DNA was checked on a Nanodrop (Thermo Scientific).
Hyas araneus	Specimens used for extraction were collected at Helgoland and Spitzbergen (Ny Ålesund). Tissue was extracted from legs using the Qiagen DNA Blood and Tissue kit according to manufacturer's protocol. Quality and quantity of DNA was checked on a Nanodrop (Thermo Scientific).
Metopaulias depressus	Sampling information: NR-15-7, Limetreegarden (Jamaica, 22.02.2009, Rivera, Schubart, Lopez, Stemmer), 18° 20,138' N - 77° 23,762' W. DNA was extracted from muscle tissue using the Qiagen DNA Blood and Tissue kit according to the manufacturer's recommendations.
Sericostoma personatum	Sampling location: Breitenbach, Hessen. Tissue was extracted from muscle tissue in the pro-, meso-, and metathorax using Qiagen DNA Blood & Tissue kit according to the manufacturer's protocol. Only 100 ul AE buffer was used for elution. Integrity of DNA was verified on a 1.5% TBE agarose gel and concentration determined on a Implen photometric cell.
Lepetodrilus sp. nov.	Sampled during JC42 RRS James Cook NERC funded ChEsSO consortium cruise. DNA was extracted both into 200ul TE buffer using Qiagen DNeasy extraction kits, then used Illustra GenomiPhi whole genome amplification to increase copy numbers, for a final concentration of 490ng/ul.
Limatula hodgsoni	Sampled during ANDEEP II ANTXIX/4 RV Polarstern cruise. DNA was extracted from tissue into 200ul TE buffer using Qiagen DNeasy extraction kits, then used Illustra GenomiPhi whole genome amplification to increase copy numbers, for a final concentration of 190ng/ul.
Arctica islandica	Specimens Ais F100 from Norway, AisF129 Kattegatt, AisF200 Iceland and AisF244from the WhiteSea were processed. Tissue was extracted from legs using the Qiagen DNA Blood and Tissue kit according to manufacturer's protocol. Quality and quantity of DNA was checked on a Nanodrop (Thermo Scientific). 1.25 microgram of each specimen was pooled.
Gorgonocephalus chilensis	The specimen of *Gorgonocephalus* was caught using Agazzis trawls from the RRS James Clark Ross from either the JR144, 179 or 230 cruises. DNA extraction was 'Salting Out' (Sunnucks and Hales 1996).
Lissarca notorcadensis	Specimens of *Lissarca notorcadenis* were caught using Agazzis trawls from the RRS James Clark Ross from the JR144 cruise. DNA extraction was 'Salting Out' (Sunnucks and Hales 1996).
Odontaster validus	Specimens of *Odontaster* were hand collected at Rothera station. DNA extraction was 'Salting Out' (Sunnucks and Hales 1996).
