# Supplementary figures and images for: Exploring Pandora's Box: Potential and Pitfalls of Low Coverage Genome Surveys for Evolutionary Biology
Source: PLoS One. 2012 Nov 21;7(11):e49202. doi: 10.1371/journal.pone.0049202 (PMC3504011; doi:10.1371/journal.pone.0049202)

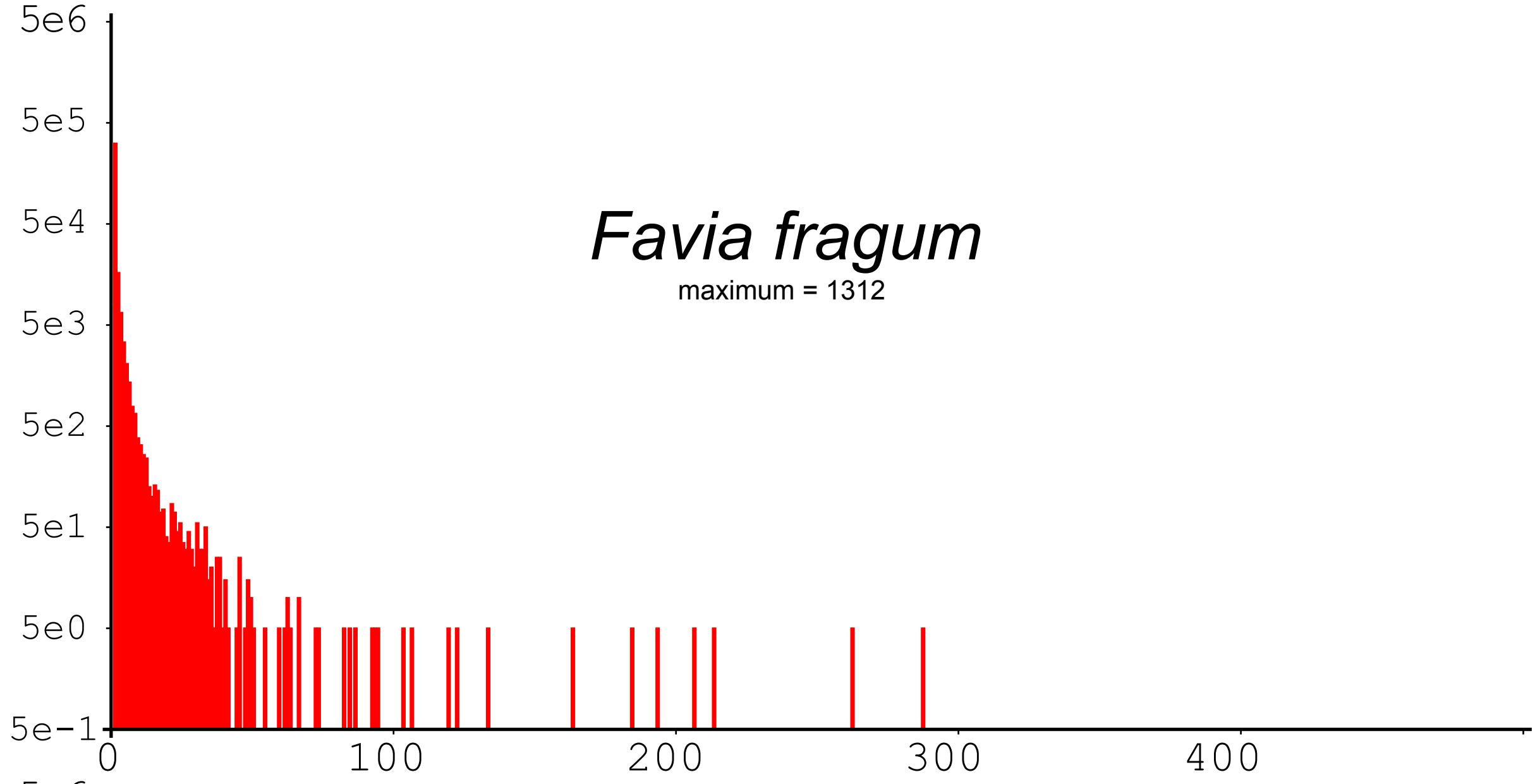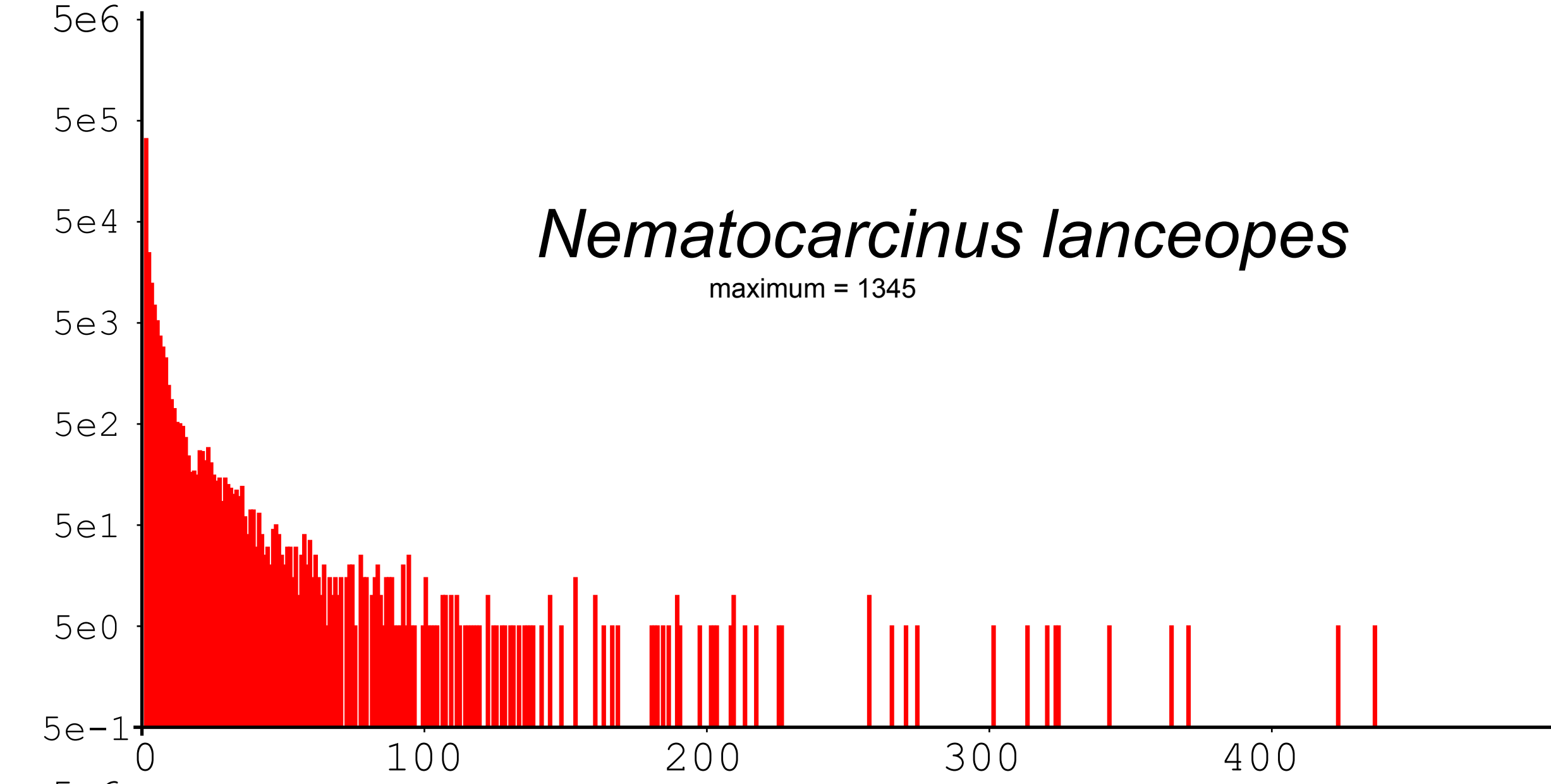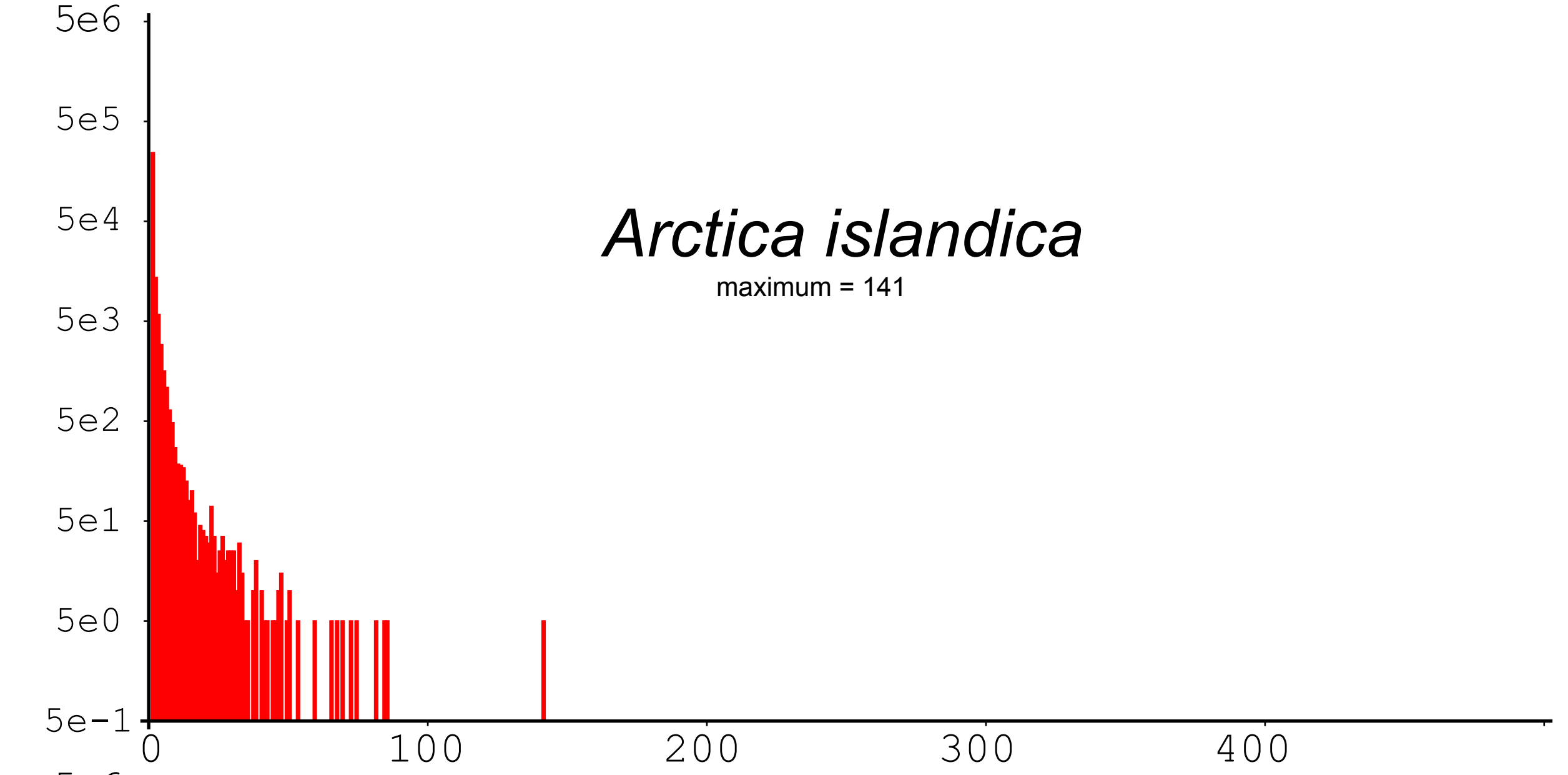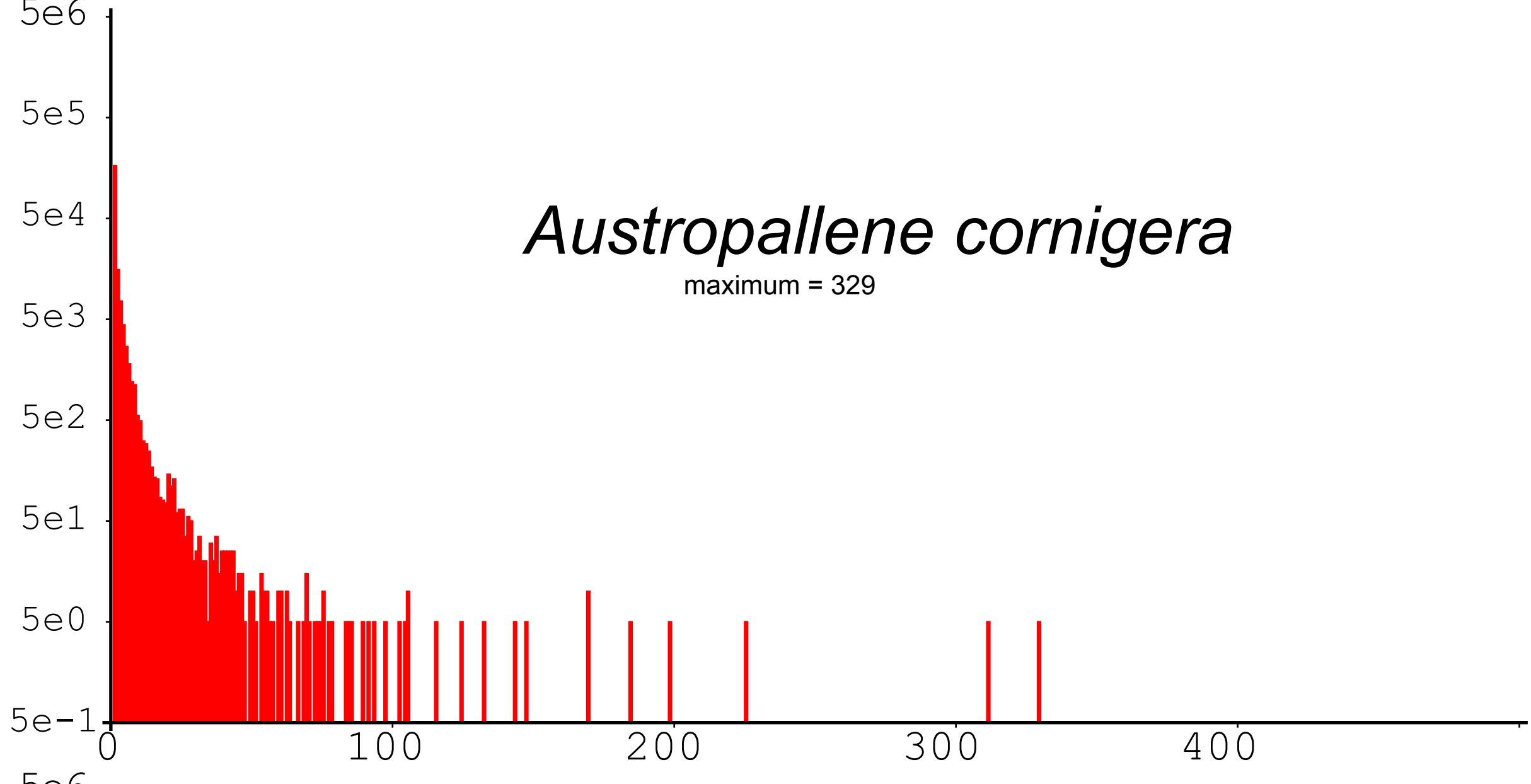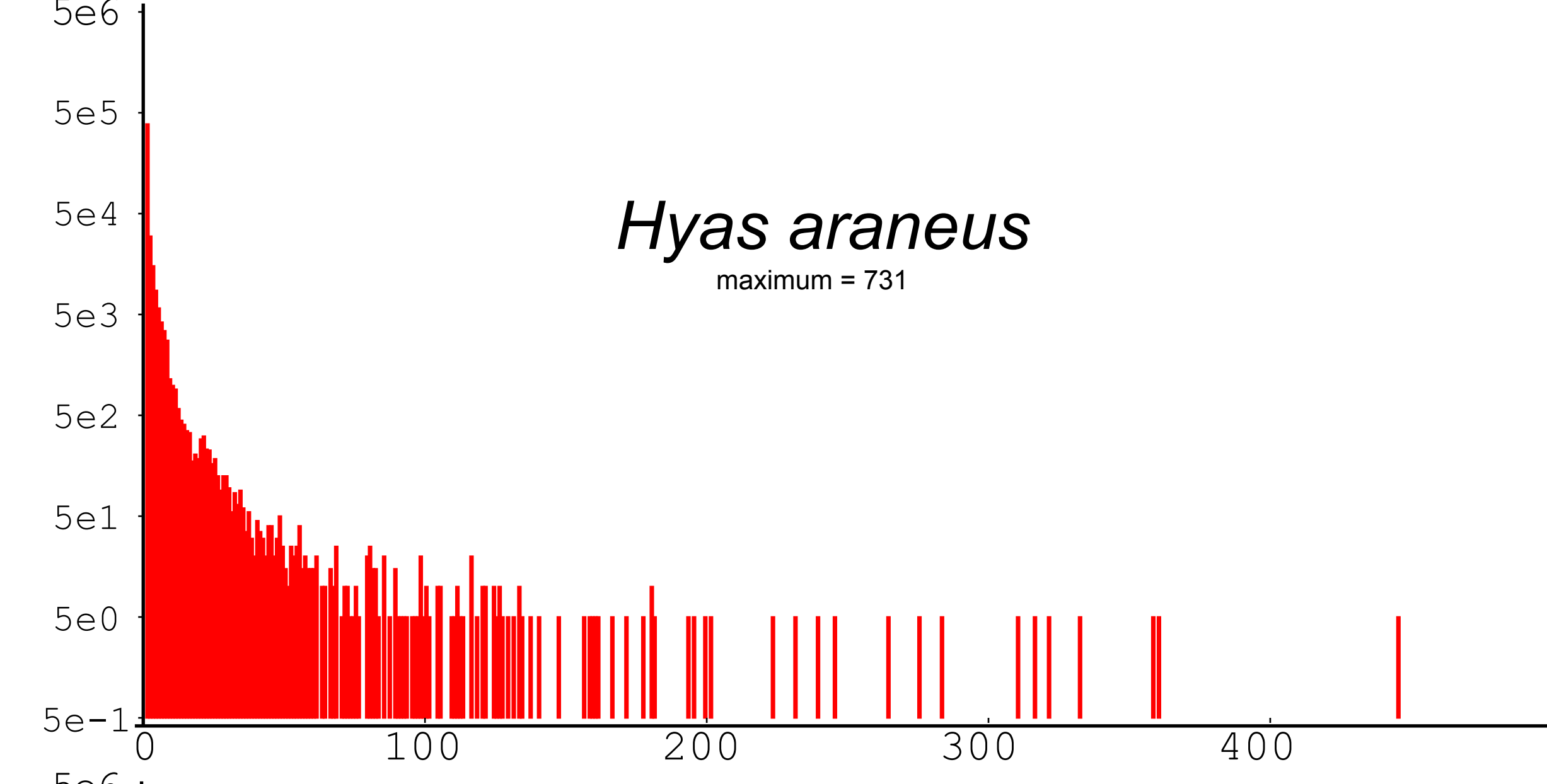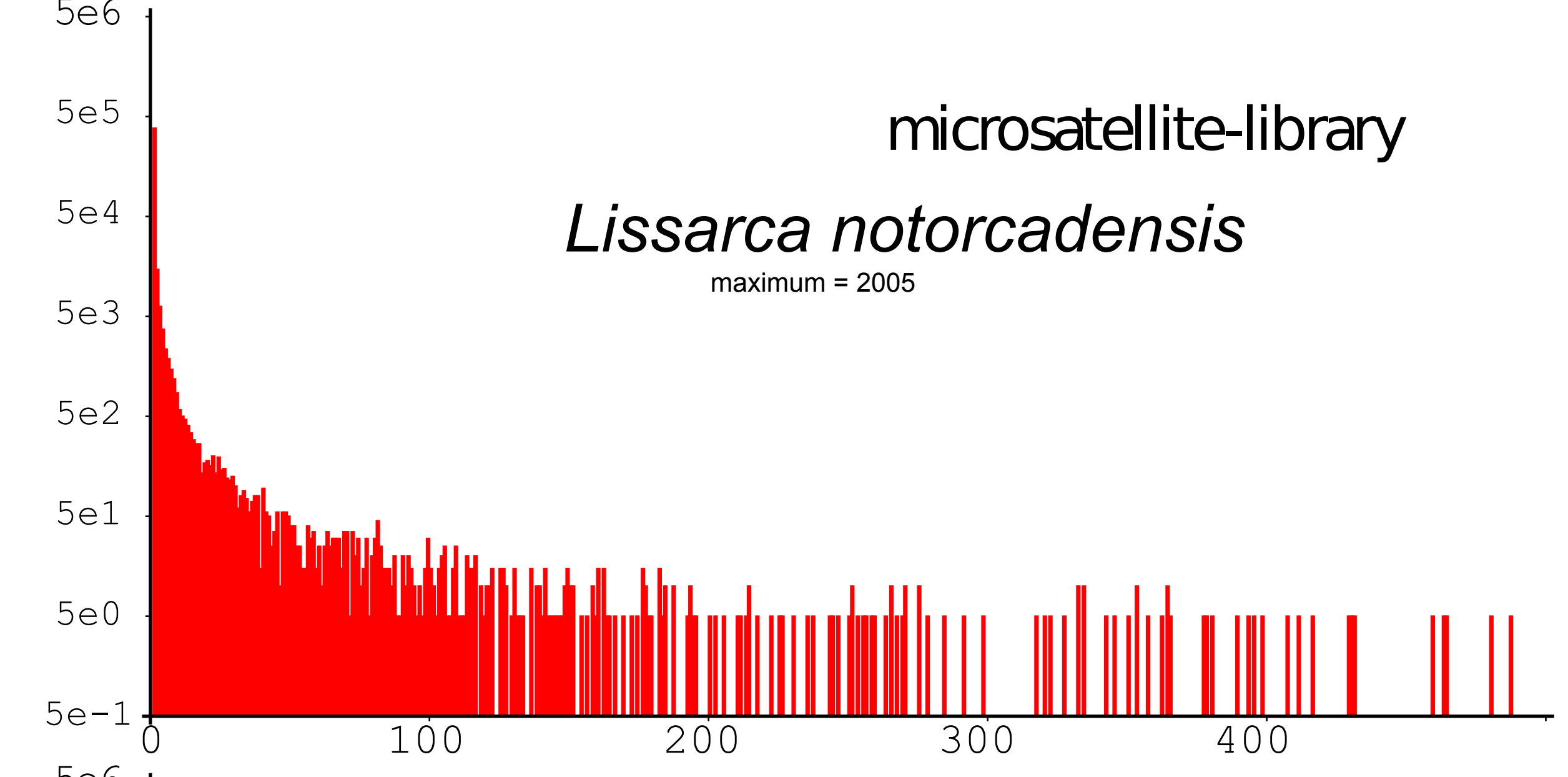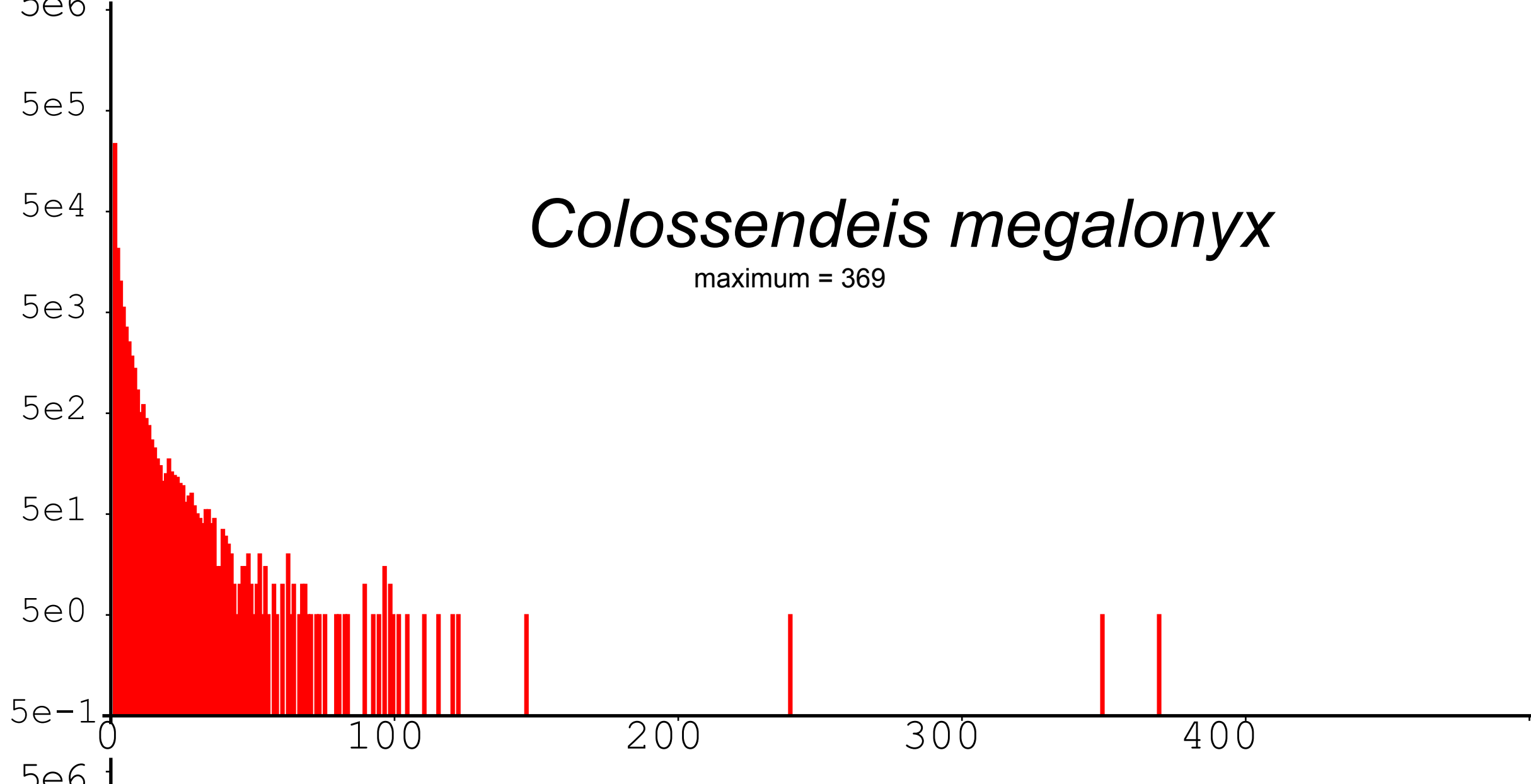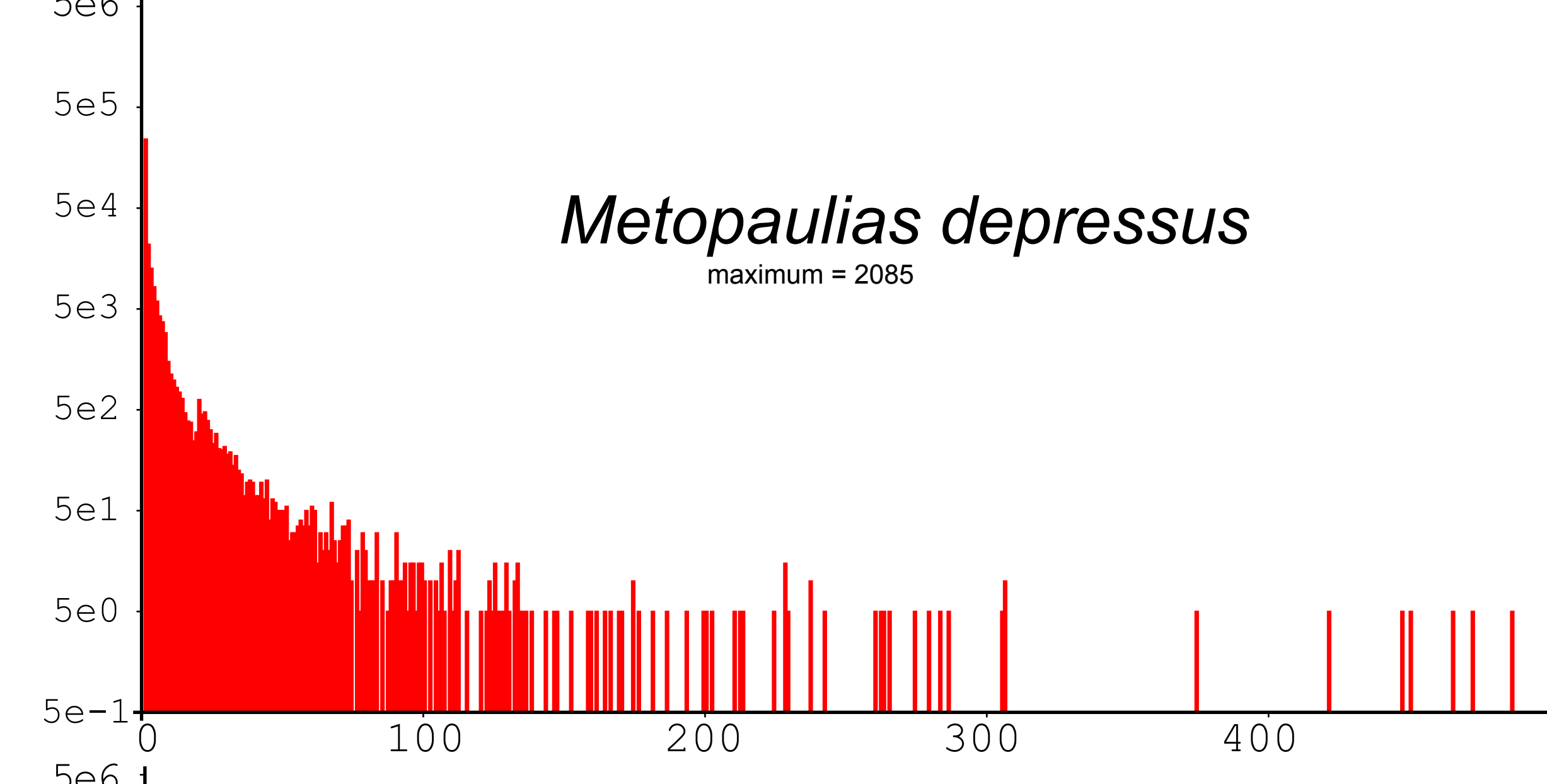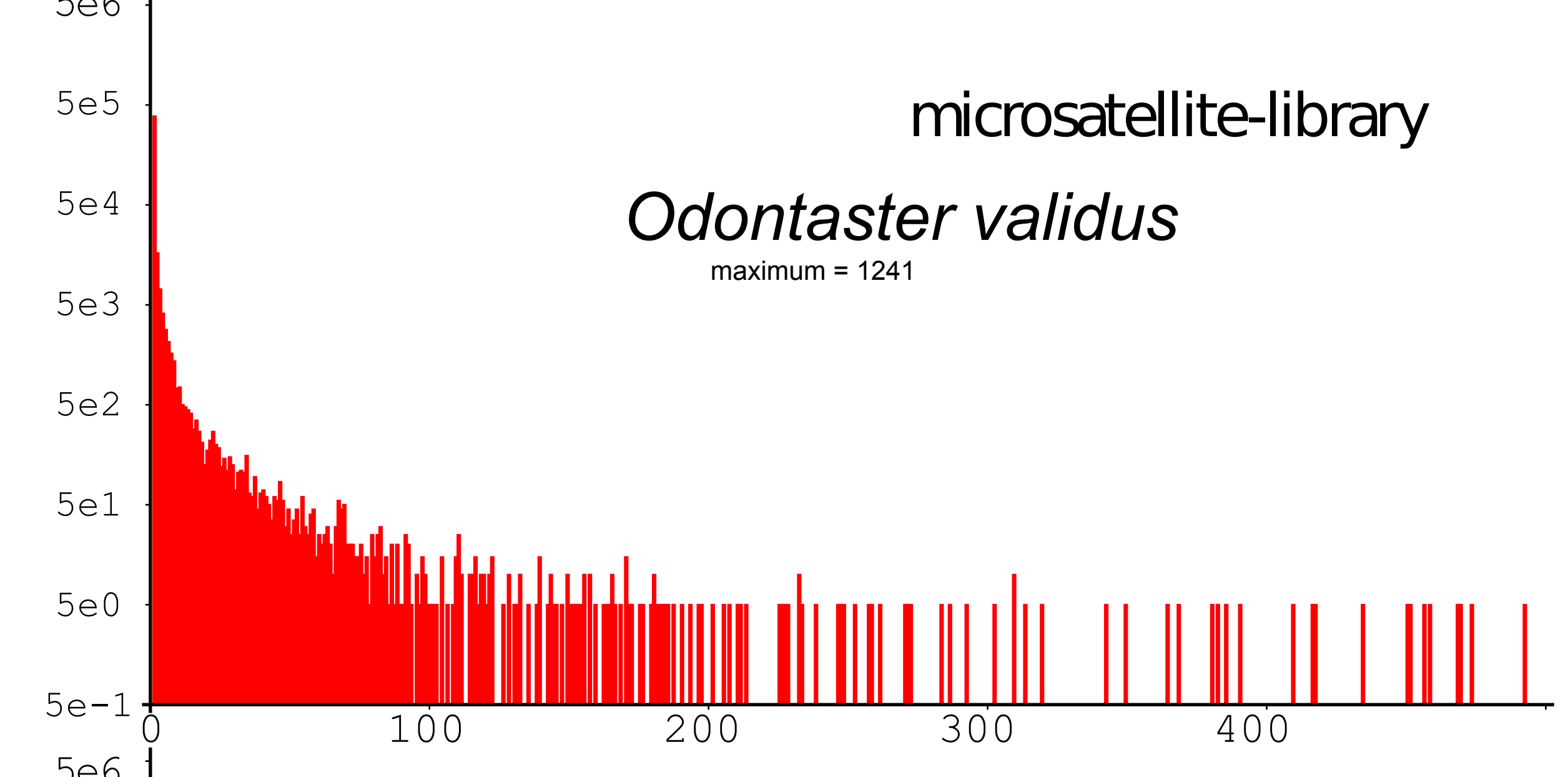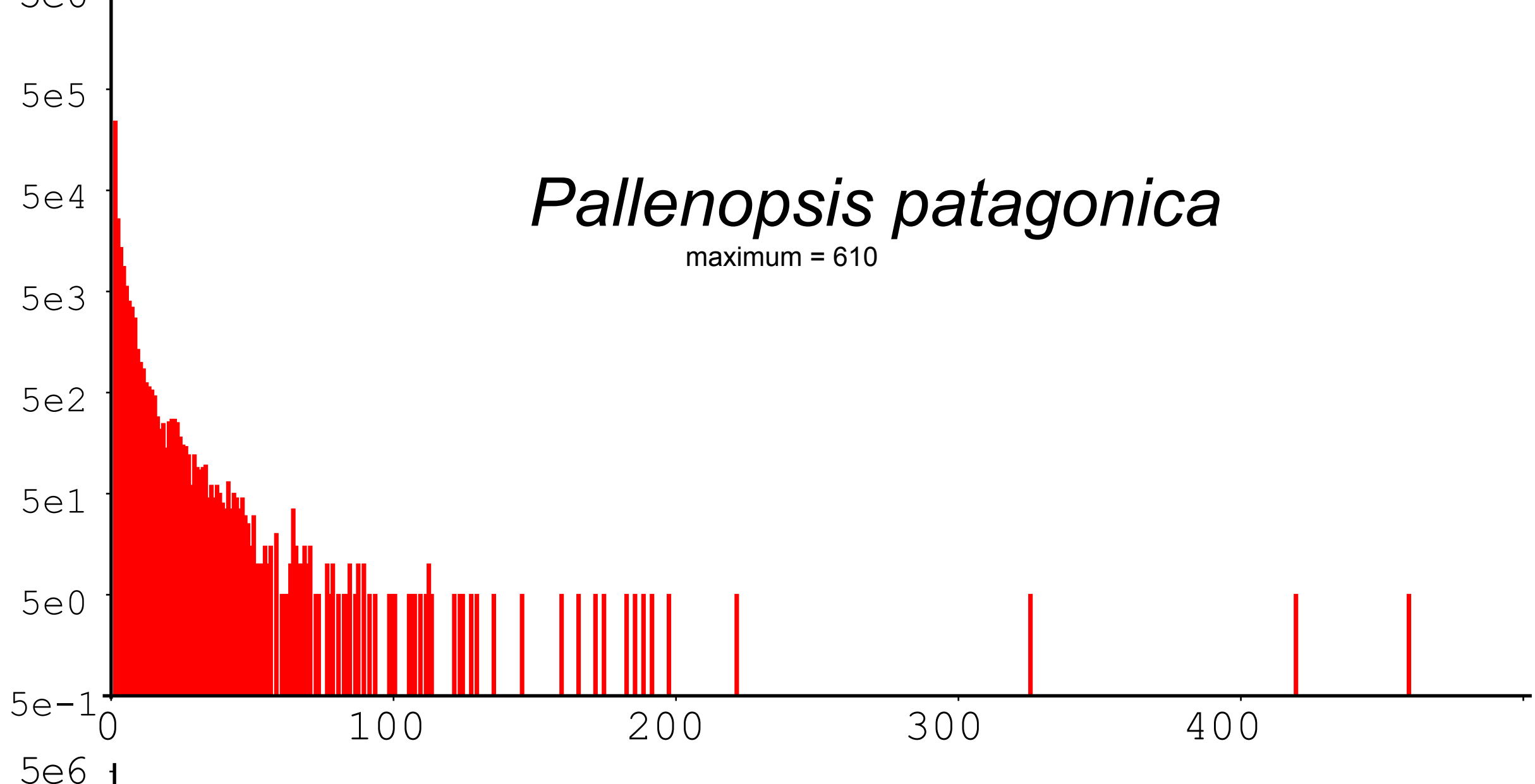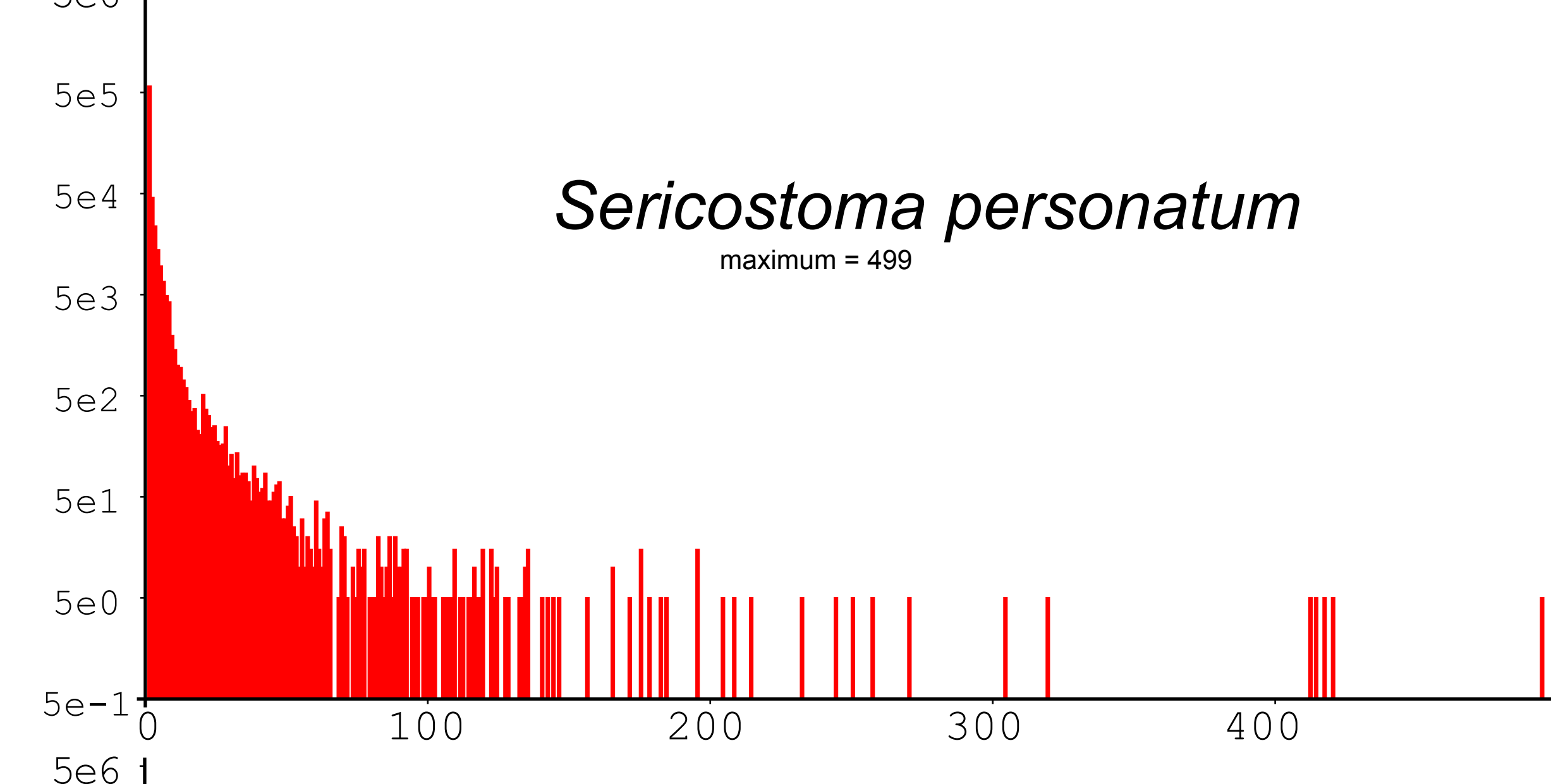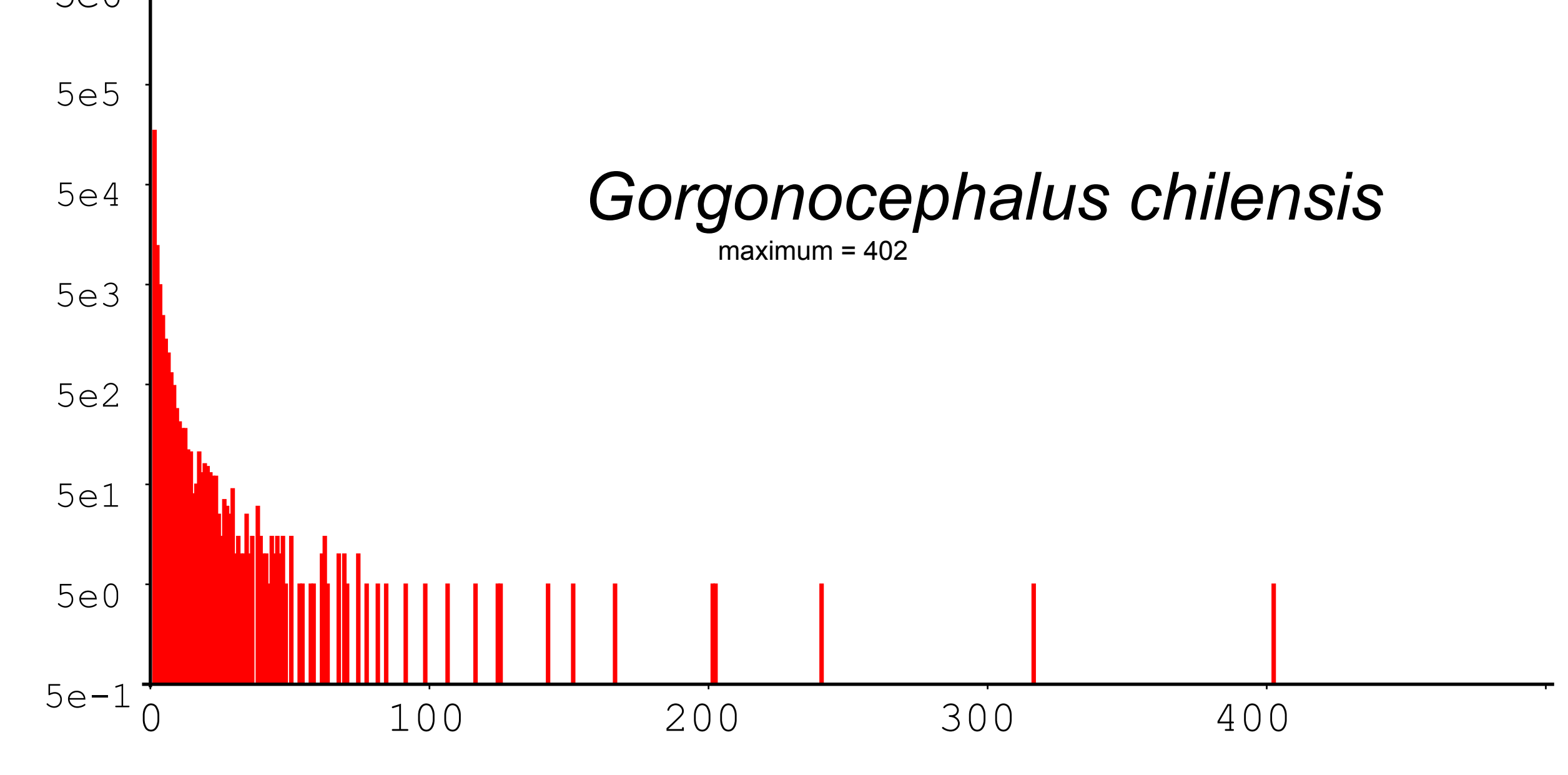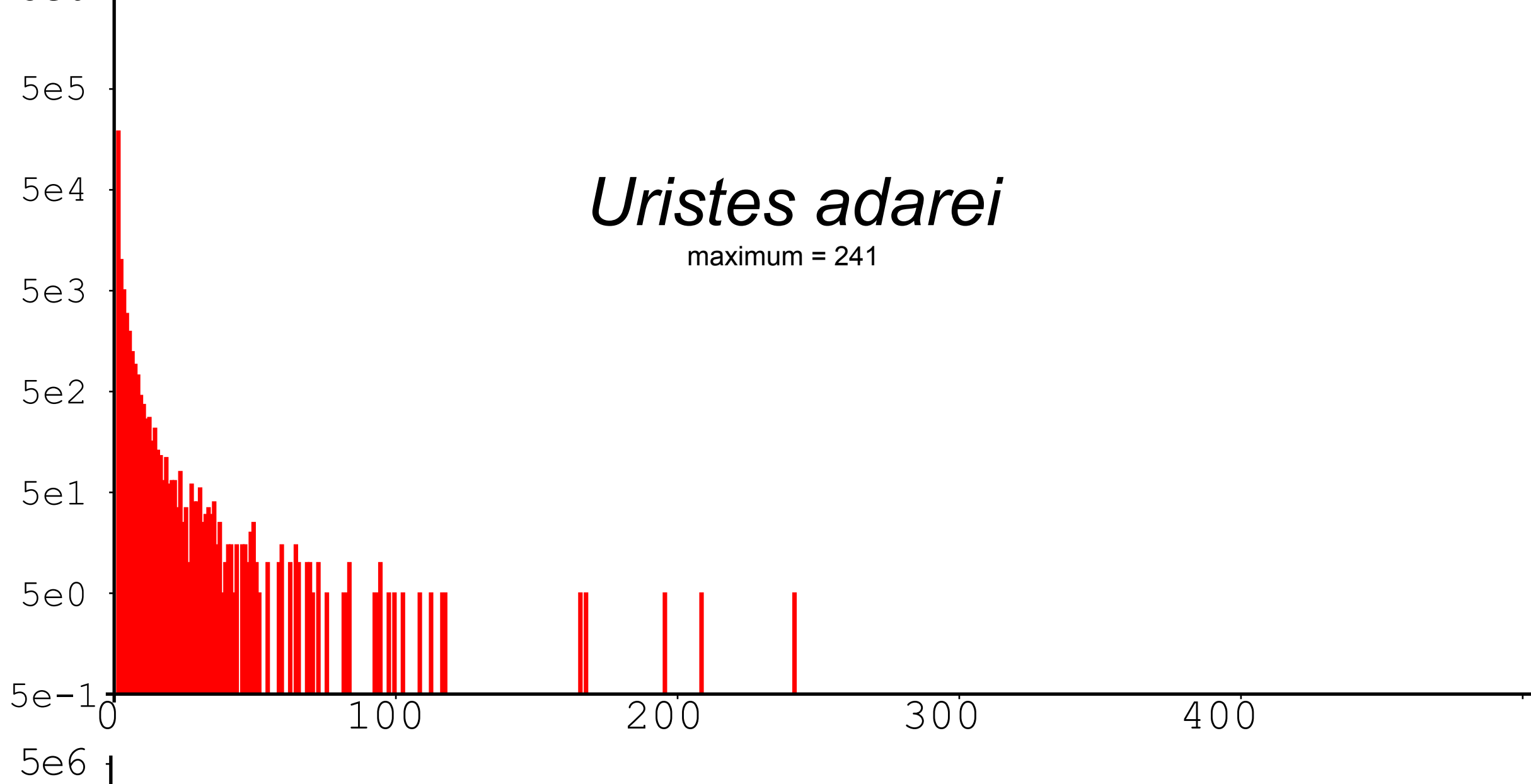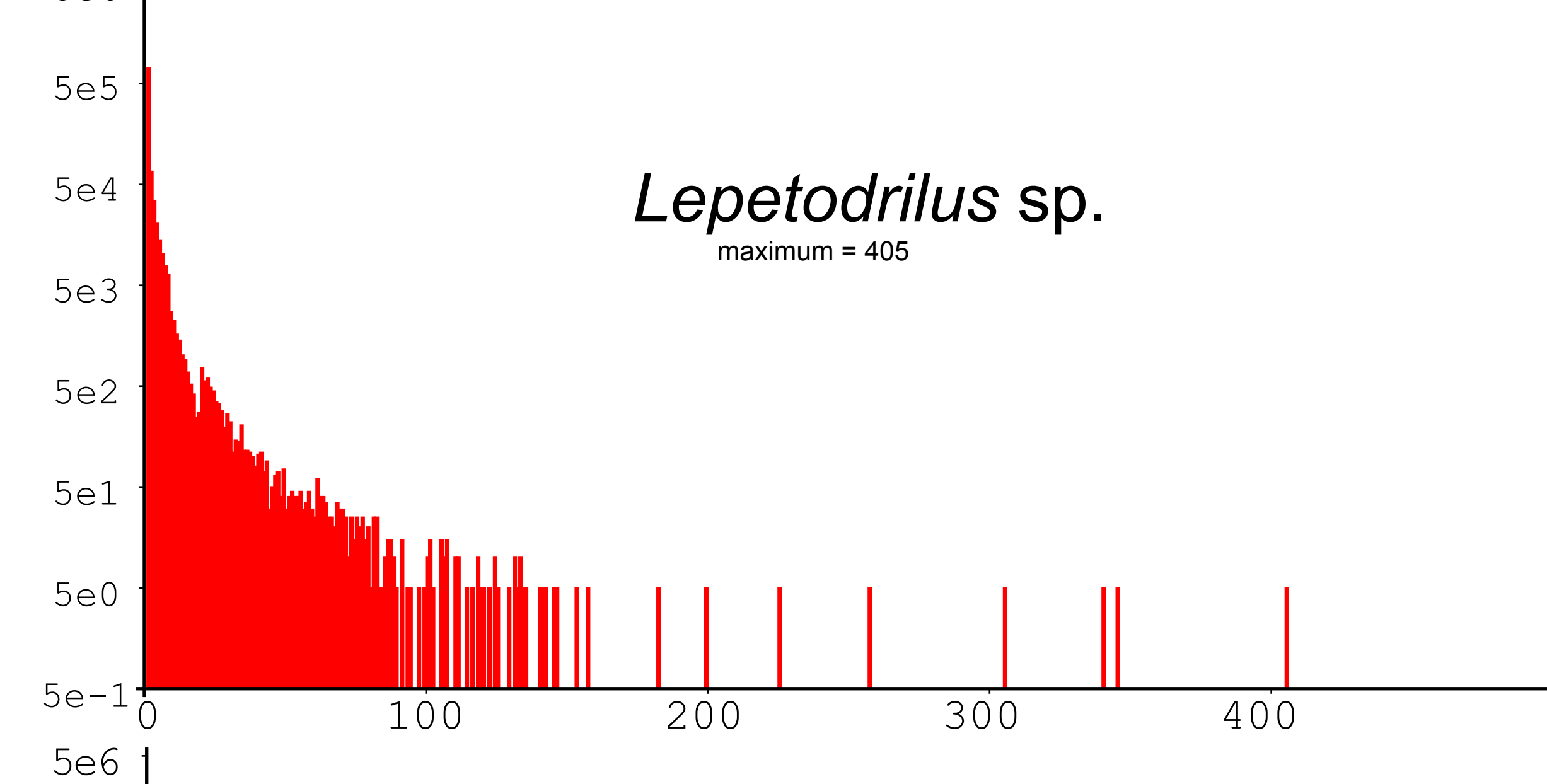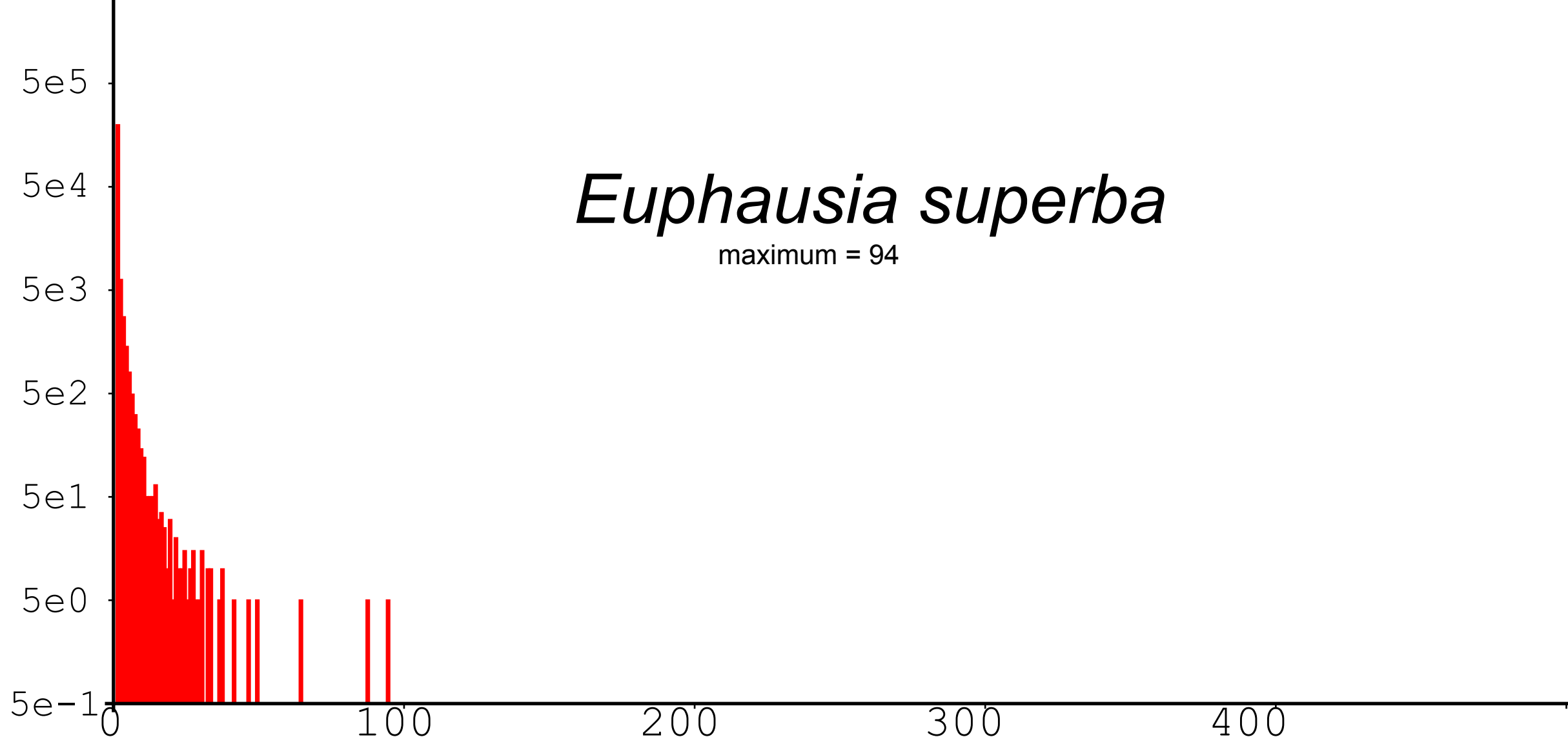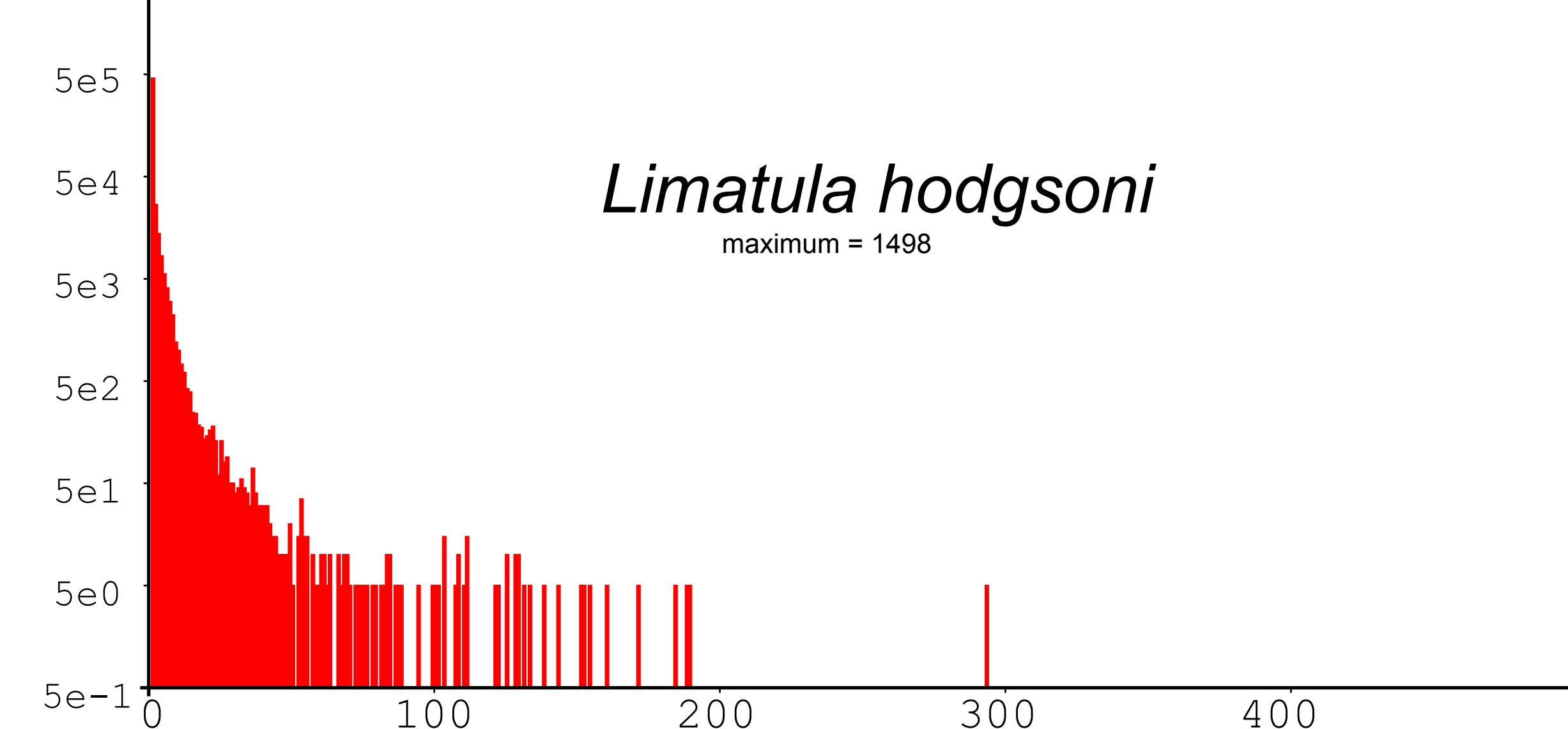

Supplement: Supporting information S2 — Overview over the assembly results for the different genomic libraries. The number of contigs (y-axis, log-scale) with the respective number of reads included in the contig (x-axis). In all cases, single-read contigs (x = 1) represented the majority of contigs after assembly. (PDF) [file pone.0049202.s002.pdf]
