# Supplementary material for: Exploring Pandora's Box: Potential and Pitfalls of Low Coverage Genome Surveys for Evolutionary Biology
Source: PLoS One. 2012 Nov 21;7(11):e49202. doi: 10.1371/journal.pone.0049202 (PMC3504011; doi:10.1371/journal.pone.0049202)
Supplement: Supporting information S6 — Taxonomic classification of bacterial hits found within the three pycnogonids and the vent limpet Lepetodrilus sp. nov. (see also Figure 7 ). (PDF) [file pone.0049202.s006.pdf]

Supporting information 6: Taxonomic classification of bacterial hits found within the three pycnogonids and the vent limpet *Lepetodrilus* sp. nov. (see also Figure 7).

a) ***Austropallene cornigera***

Phyla list

Phyla	Reads, num	Contigs, num	Contigs, bp
Proteobacteria	1135	519	212994
Unknown bacteria	8	8	2834
Firmicutes	6	6	2347
Cyanobacteria	4	4	1982

Classes list

Phyla	Classes	Reads, num	Contigs, num	Contigs, bp
Proteobacteria	Gammaproteobacteria	1035	510	207402
Proteobacteria	Epsilonproteobacteria	89	1	2465
Proteobacteria	Alphaproteobacteria	10	7	2651
	Unknown bacteria	8	8	2834
Firmicutes	Bacilli	5	5	1936
Cyanobacteria		4	4	1982
Proteobacteria	Betaproteobacteria	1	1	476
Firmicutes	Clostridia	1	1	411

Genera list

Phyla	Classes	Genera	Reads, num	Contigs, num	Contigs, bp
Proteobacteria	Gammaproteobacteria	Psychromonas	317	175	68531
Proteobacteria	Gammaproteobacteria	Shewanella	239	61	27793
Proteobacteria	Epsilonproteobacteria	Helicobacter	89	1	2465
Proteobacteria	Gammaproteobacteria	Aliivibrio	88	59	22053
Proteobacteria	Gammaproteobacteria	Pseudoalteromonas	83	35	15875
Proteobacteria	Gammaproteobacteria	Colwellia	62	27	11162
Proteobacteria	Gammaproteobacteria	Photobacterium	60	31	13195
Proteobacteria	Gammaproteobacteria	Vibrio	32	21	7789
Proteobacteria	Gammaproteobacteria	Glaciecola	25	12	5174
Proteobacteria	Gammaproteobacteria	Haemophilus	22	18	6790
Proteobacteria	Gammaproteobacteria	Acinetobacter	10	8	3550
Proteobacteria	Gammaproteobacteria	Gammaproteobacteria	10	10	3933
Proteobacteria	Gammaproteobacteria	Proteus	9	4	1284
Proteobacteria	Gammaproteobacteria	Marinomonas	9	6	2442
Proteobacteria	Gammaproteobacteria	Xenorhabdus	8	5	1771

Proteobacteria	Gammaproteobacteria	Serratia	8	1	971
		Unknown bacteria	8	8	2834
Proteobacteria	Gammaproteobacteria	Listonella	5	4	1462
Proteobacteria	Gammaproteobacteria	Candidatus Regiella	5	1	918
Proteobacteria	Gammaproteobacteria	Rahnella	5	1	524
Proteobacteria	Gammaproteobacteria	Candidatus Hamiltonella	5	1	475
Proteobacteria	Gammaproteobacteria	Photorhabdus	4	4	1662
Cyanobacteria		Synechococcus	4	4	1982
Proteobacteria	Gammaproteobacteria	Actinobacillus	4	2	463
Proteobacteria	Gammaproteobacteria	Histophilus	4	4	1753
Proteobacteria	Gammaproteobacteria	Aggregatibacter	3	3	1458
Proteobacteria	Gammaproteobacteria	Gallibacterium	3	2	772
Proteobacteria	Alphaproteobacteria	Oligotropha	3	1	440
Proteobacteria	Alphaproteobacteria	Bartonella	2	1	137
Proteobacteria	Gammaproteobacteria	Salmonella	2	2	918
Firmicutes	Bacilli	Staphylococcus	2	2	940
Proteobacteria	Gammaproteobacteria	Pantoea	2	2	783
Proteobacteria	Gammaproteobacteria	Pasteurella	2	2	924
Proteobacteria	Alphaproteobacteria	Phenylobacterium	1	1	457
Proteobacteria	Gammaproteobacteria	Enterobacter	1	1	54
Proteobacteria	Gammaproteobacteria	Erwinia	1	1	225
Firmicutes	Bacilli	Listeria	1	1	48
Proteobacteria	Betaproteobacteria	Nitrosomonas	1	1	476
Proteobacteria	Gammaproteobacteria	Alteromonas	1	1	61
Firmicutes	Bacilli	Bacillus	1	1	431
Proteobacteria	Gammaproteobacteria	Aeromonas	1	1	500
Proteobacteria	Alphaproteobacteria	Roseobacter	1	1	359
Firmicutes	Clostridia	Clostridium	1	1	411
Proteobacteria	Alphaproteobacteria	Rickettsia	1	1	491
Proteobacteria	Gammaproteobacteria	Citrobacter	1	1	454
Proteobacteria	Alphaproteobacteria	Hirschia	1	1	340
Proteobacteria	Gammaproteobacteria	Mannheimia	1	1	425
Proteobacteria	Gammaproteobacteria	Moritella	1	1	450
Firmicutes	Bacilli	Geobacillus	1	1	517
Proteobacteria	Gammaproteobacteria	Francisella	1	1	317
Proteobacteria	Gammaproteobacteria	Kangiella	1	1	491
Proteobacteria	Alphaproteobacteria	Rhizobium	1	1	427

Species list

Phyla	Classes	Species	Reads,	Contigs,	Contigs,

			num	num	bp
Proteobacteria	Gammaproteobacteria	Psychromonas ingrahamii	307	174	67202
Proteobacteria	Epsilonproteobacteria	Helicobacter pylori	89	1	2465
Proteobacteria	Gammaproteobacteria	Shewanella pealeana	79	10	5226
Proteobacteria	Gammaproteobacteria	Shewanella oneidensis	74	6	4725
Proteobacteria	Gammaproteobacteria	Colwellia psychrerythraea	62	27	11162
Proteobacteria	Gammaproteobacteria	Photobacterium profundum	60	31	13195
Proteobacteria	Gammaproteobacteria	Aliivibrio salmonicida	51	32	11716
Proteobacteria	Gammaproteobacteria	Aliivibrio fischeri	37	27	10337
Proteobacteria	Gammaproteobacteria	Shewanella piezotolerans	29	10	3702
Proteobacteria	Gammaproteobacteria	Pseudoalteromonas sp. SM9913	29	17	6375
Proteobacteria	Gammaproteobacteria	Pseudoalteromonas atlantica	29	6	3564
Proteobacteria	Gammaproteobacteria	Pseudoalteromonas haloplanktis	25	12	5936
Proteobacteria	Gammaproteobacteria	Glaciecola sp. 4H-3-7+YE-5	25	12	5174
Proteobacteria	Gammaproteobacteria	Shewanella frigidimarina	18	11	3967
Proteobacteria	Gammaproteobacteria	Vibrio splendidus	10	6	2037
Proteobacteria	Gammaproteobacteria	Psychromonas sp. BSw20972	10	1	1329
Proteobacteria	Gammaproteobacteria	Shewanella putrefaciens	9	2	911
Proteobacteria	Gammaproteobacteria	Proteus mirabilis	9	4	1284
Proteobacteria	Gammaproteobacteria	Haemophilus ducreyi	8	8	3130
Proteobacteria	Gammaproteobacteria	Serratia proteamaculans	8	1	971
Proteobacteria	Gammaproteobacteria	Xenorhabdus bovienii	8	5	1771
Proteobacteria	Gammaproteobacteria	Haemophilus parainfluenzae	7	6	1827
		uncultured marine bacterium	6	6	2336
Proteobacteria	Gammaproteobacteria	Marinomonas mediterranea	6	3	1209
Proteobacteria	Gammaproteobacteria	Haemophilus influenzae	6	3	1498
Proteobacteria	Gammaproteobacteria	Shewanella sediminis	6	3	1586
Proteobacteria	Gammaproteobacteria	Rahnella sp. Y9602	5	1	524
Proteobacteria	Gammaproteobacteria	Candidatus Hamiltonella defensa	5	1	475
Proteobacteria	Gammaproteobacteria	Vibrio parahaemolyticus	5	3	1216
Proteobacteria	Gammaproteobacteria	Shewanella sp. W3-18-1	5	1	518
Proteobacteria	Gammaproteobacteria	uncultured marine gamma proteobacterium EBAC20E09	5	5	1927
Proteobacteria	Gammaproteobacteria	Candidatus Regiella insecticola	5	1	918
Proteobacteria	Gammaproteobacteria	Vibrio harveyi	5	4	1381
Proteobacteria	Gammaproteobacteria	Shewanella baltica	5	4	1048
Proteobacteria	Gammaproteobacteria	Listonella anguillarum	5	4	1462
Proteobacteria	Gammaproteobacteria	Vibrio vulnificus	5	2	802
Proteobacteria	Gammaproteobacteria	Acinetobacter baumannii	4	2	736
Proteobacteria	Gammaproteobacteria	Actinobacillus pleuropneumoniae	4	2	463

Proteobacteria	Gammaproteobacteria	Histophilus somni	4	4	1753
Cyanobacteria		Synechococcus sp. PCC 7002	4	4	1982
Proteobacteria	Gammaproteobacteria	Shewanella violacea	4	4	1404
Proteobacteria	Gammaproteobacteria	Shewanella sp. ANA-3	3	3	1370
Proteobacteria	Gammaproteobacteria	Acinetobacter calcoaceticus	3	3	1597
Proteobacteria	Gammaproteobacteria	Shewanella halifaxensis	3	3	1362
Proteobacteria	Gammaproteobacteria	Photorhabdus asymbiotica	3	3	1159
Proteobacteria	Gammaproteobacteria	Aggregatibacter actinomycetemcomitans	3	3	1458
Proteobacteria	Gammaproteobacteria	Gallibacterium anatis	3	2	772
Proteobacteria	Alphaproteobacteria	Oligotropha carboxidovorans	3	1	440
Proteobacteria	Gammaproteobacteria	Marinomonas sp. MWYL1	3	3	1233
Proteobacteria	Gammaproteobacteria	Candidatus Baumannia cicadellinicola	2	2	849
Proteobacteria	Gammaproteobacteria	Vibrio cincinnatiensis	2	1	263
Proteobacteria	Gammaproteobacteria	Salmonella enterica	2	2	918
Proteobacteria	Gammaproteobacteria	Vibrio furnissii	2	2	874
Proteobacteria	Gammaproteobacteria	Calyptogena okutanii thioautotrophic gill symbiont	2	2	875
Proteobacteria	Alphaproteobacteria	Bartonella quintana	2	1	137
Proteobacteria	Gammaproteobacteria	Acinetobacter sp. HY-7	2	2	703
Proteobacteria	Gammaproteobacteria	Shewanella woodyi	2	2	952
Proteobacteria	Gammaproteobacteria	Pasteurella multocida	2	2	924
Firmicutes	Bacilli	Staphylococcus aureus	2	2	940
Proteobacteria	Alphaproteobacteria	Phenylobacterium zucineum	1	1	457
Firmicutes	Bacilli	Listeria monocytogenes	1	1	48
Proteobacteria	Gammaproteobacteria	Enterobacter aerogenes	1	1	54
		uncultured bacterium CBNPD1 BAC clone 2089	1	1	454
Proteobacteria	Gammaproteobacteria	Aeromonas salmonicida	1	1	500
Proteobacteria	Gammaproteobacteria	Erwinia amylovora	1	1	225
Proteobacteria	Alphaproteobacteria	Roseobacter litoralis	1	1	359
Proteobacteria	Betaproteobacteria	Nitrosomonas eutropha	1	1	476
Firmicutes	Bacilli	Bacillus cellulosilyticus	1	1	431
Proteobacteria	Gammaproteobacteria	Alteromonas macleodii	1	1	61
Proteobacteria	Gammaproteobacteria	Shewanella loihica	1	1	510
Proteobacteria	Gammaproteobacteria	gamma proteobacterium HdN1	1	1	282
Proteobacteria	Gammaproteobacteria	Shewanella denitrificans	1	1	512
Proteobacteria	Gammaproteobacteria	Haemophilus parasuis	1	1	335
Proteobacteria	Alphaproteobacteria	Rhizobium leguminosarum	1	1	427
Proteobacteria	Gammaproteobacteria	Photorhabdus luminescens	1	1	503

Proteobacteria	Alphaproteobacteria	Rickettsia peacockii	1	1	491
Proteobacteria	Gammaproteobacteria	Vibrio sp. Ex25	1	1	446
Proteobacteria	Gammaproteobacteria	Pantoea sp. At-9b	1	1	358
		uncultured bacterium	1	1	44
Proteobacteria	Gammaproteobacteria	Citrobacter rodentium	1	1	454
Proteobacteria	Gammaproteobacteria	Pantoea vagans	1	1	425
Proteobacteria	Gammaproteobacteria	Acinetobacter sp. DR1	1	1	514
Proteobacteria	Gammaproteobacteria	Mannheimia haemolytica	1	1	425
Proteobacteria	Gammaproteobacteria	Moritella marina	1	1	450
Proteobacteria	Alphaproteobacteria	Hirschia baltica	1	1	340
Firmicutes	Bacilli	Geobacillus thermodenitrificans	1	1	517
Proteobacteria	Gammaproteobacteria	Francisella sp. TX077308	1	1	317
Proteobacteria	Gammaproteobacteria	Kangiella koreensis	1	1	491
Proteobacteria	Gammaproteobacteria	Vibrio mimicus	1	1	442
Firmicutes	Clostridia	Clostridium phytofermentans	1	1	411
Proteobacteria	Gammaproteobacteria	Vibrio pacinii	1	1	328

b) ***Pallenopsis patagonica***

Phyla list

Phyla	Reads, num	Contigs, num	Contigs, bp
Proteobacteria	56	52	20757
Unknown bacteria	2	2	556

Classes list

Phyla	Classes	Reads, num	Contigs, num	Contigs, bp
Proteobacteria	Gammaproteobacteria	47	43	17642
Proteobacteria	Alphaproteobacteria	6	6	2393
Proteobacteria	Betaproteobacteria	3	3	722
	Unknown bacteria	2	2	556

Genera list

Phyla	Classes	Genera	Reads, num	Contigs, num	Contigs, bp
Proteobacteria	Gammaproteobacteria	Colwellia	17	17	7325
Proteobacteria	Gammaproteobacteria	Pseudoalteromonas	11	11	3769
Proteobacteria	Gammaproteobacteria	Psychrobacter	5	1	573
Proteobacteria	Gammaproteobacteria	Photobacterium	5	5	2298
Proteobacteria	Betaproteobacteria	Ralstonia	3	3	722
Proteobacteria	Gammaproteobacteria	Saccharophagus	2	2	978
Proteobacteria	Gammaproteobacteria	Aliivibrio	2	2	876
		Unknown bacteria	2	2	556
Proteobacteria	Alphaproteobacteria	Ruegeria	2	2	870
Proteobacteria	Gammaproteobacteria	Alteromonas	1	1	241
Proteobacteria	Gammaproteobacteria	Salmonella	1	1	426
Proteobacteria	Alphaproteobacteria	Rhodopseudomonas	1	1	408
Proteobacteria	Gammaproteobacteria	Vibrio	1	1	401
Proteobacteria	Alphaproteobacteria	Mesorhizobium	1	1	273
Proteobacteria	Gammaproteobacteria	Moritella	1	1	344
Proteobacteria	Alphaproteobacteria	Rhodobacter	1	1	389
Proteobacteria	Gammaproteobacteria	Marinobacter	1	1	411
Proteobacteria	Alphaproteobacteria	Agrobacterium	1	1	453

Species list

Phyla	Classes	Species	Reads, num	Contigs, num	Contigs, bp
Proteobacteria	Gammaproteobacteria	Colwellia psychrerythraea	17	17	7325
Proteobacteria	Gammaproteobacteria	Pseudoalteromonas haloplanktis	10	10	3316
Proteobacteria	Gammaproteobacteria	Psychrobacter cryohalolentis	5	1	573

Proteobacteria	Gammaproteobacteria	Photobacterium profundum	5	5	2298
Proteobacteria	Betaproteobacteria	Ralstonia pickettii	3	3	722
Proteobacteria	Gammaproteobacteria	Saccharophagus degradans	2	2	978
Proteobacteria	Alphaproteobacteria	Ruegeria pomeroyi	2	2	870
Proteobacteria	Gammaproteobacteria	Aliivibrio salmonicida	2	2	876
		uncultured bacterium ARCTIC03_F_03	1	1	482
Proteobacteria	Gammaproteobacteria	Salmonella enterica	1	1	426
Proteobacteria	Gammaproteobacteria	Alteromonas sp. SN2	1	1	241
		uncultured bacterium	1	1	74
Proteobacteria	Alphaproteobacteria	Rhodopseudomonas palustris	1	1	408
Proteobacteria	Gammaproteobacteria	Vibrio splendidus	1	1	401
Proteobacteria	Gammaproteobacteria	Pseudoalteromonas sp. SM9913	1	1	453
Proteobacteria	Alphaproteobacteria	Mesorhizobium opportunistum	1	1	273
Proteobacteria	Gammaproteobacteria	Moritella profunda	1	1	344
Proteobacteria	Alphaproteobacteria	Rhodobacter sphaeroides	1	1	389
Proteobacteria	Gammaproteobacteria	Marinobacter hydrocarbonoclasticus	1	1	411
Proteobacteria	Alphaproteobacteria	Agrobacterium vitis	1	1	453

c) ***Colossendeis megalonyx***

Phyla list

Phyla	Reads, num	Contigs, num	Contigs, bp
Proteobacteria	132	130	50768
Tenericutes	40	31	14204
Thermotogae	4	4	1938
Firmicutes	2	2	1001
Actinobacteria	2	2	929
Bacteroidetes	1	1	409

Classes list

Phyla	Classes	Reads, num	Contigs, num	Contigs, bp
Proteobacteria	Gammaproteobacteria	64	62	22713
Proteobacteria	Betaproteobacteria	50	50	21564
Tenericutes	Mollicutes	40	31	14204
Proteobacteria	Alphaproteobacteria	18	18	6491
Thermotogae	Thermotogae	4	4	1938
Actinobacteria	Actinobacteria	2	2	929
Firmicutes	Clostridia	1	1	479
Bacteroidetes	Flavobacteria	1	1	409
Firmicutes	Bacilli	1	1	522

Genera list

Phyla	Classes	Genera	Reads, num	Contigs, num	Contigs, bp
Proteobacteria	Betaproteobacteria	Ralstonia	40	40	18012
Tenericutes	Mollicutes	Mycoplasma	39	30	14146
Proteobacteria	Gammaproteobacteria	Stenotrophomonas	30	30	10870
Proteobacteria	Gammaproteobacteria	Serratia	21	21	7840
Proteobacteria	Alphaproteobacteria	Brevundimonas	14	14	5162
Proteobacteria	Gammaproteobacteria	Escherichia	9	7	2512
Thermotogae	Thermotogae	Thermosipho	4	4	1938
Proteobacteria	Betaproteobacteria	Cupriavidus	4	4	1503
Proteobacteria	Betaproteobacteria	Candidatus Accumulibacter	2	2	768
Proteobacteria	Betaproteobacteria	Achromobacter	2	2	774
Actinobacteria	Actinobacteria	Propionibacterium	2	2	929
Proteobacteria	Betaproteobacteria	Bordetella	1	1	40
Bacteroidetes	Flavobacteria	Flavobacterium	1	1	409
Proteobacteria	Gammaproteobacteria	Buchnera	1	1	498
Firmicutes	Clostridia	Alkaliphilus	1	1	479

Tenericutes	Mollicutes	Mesoplasma	1	1	58
Proteobacteria	Alphaproteobacteria	Caulobacter	1	1	192
Proteobacteria	Gammaproteobacteria	Xanthomonas	1	1	45
Proteobacteria	Betaproteobacteria	Burkholderia	1	1	467
Proteobacteria	Gammaproteobacteria	Pseudomonas	1	1	461
Firmicutes	Bacilli	Lactobacillus	1	1	522
Proteobacteria	Alphaproteobacteria	Ochrobactrum	1	1	419
Proteobacteria	Alphaproteobacteria	Dinoroseobacter	1	1	472
Proteobacteria	Alphaproteobacteria	Azospirillum	1	1	246
Proteobacteria	Gammaproteobacteria	Enterobacter	1	1	487

Species list

Phyla	Classes	Species	Reads, num	Contigs, num	Contigs, bp
Proteobacteria	Betaproteobacteria	Ralstonia pickettii	38	38	17075
Proteobacteria	Gammaproteobacteria	Stenotrophomonas maltophilia	30	30	10870
Proteobacteria	Gammaproteobacteria	Serratia sp. AS13	15	15	5605
Proteobacteria	Alphaproteobacteria	Brevundimonas subvibrioides	13	13	4804
Tenericutes	Mollicutes	Mycoplasma penetrans	9	2	1221
Proteobacteria	Gammaproteobacteria	Escherichia coli	9	7	2512
Tenericutes	Mollicutes	Mycoplasma pulmonis	9	9	4531
Tenericutes	Mollicutes	Mycoplasma agalactiae	7	5	2085
Proteobacteria	Gammaproteobacteria	Serratia proteamaculans	5	5	1990
Thermotogae	Thermotogae	Thermosipho africanus	4	4	1938
Proteobacteria	Betaproteobacteria	Cupriavidus pinatubonensis	4	4	1503
Tenericutes	Mollicutes	Mycoplasma mobile	3	3	899
Proteobacteria	Betaproteobacteria	Ralstonia solanacearum	2	2	937
Tenericutes	Mollicutes	Mycoplasma iguanae	2	2	940
Tenericutes	Mollicutes	Mycoplasma crocodyli	2	2	1039
Proteobacteria	Betaproteobacteria	Candidatus Accumulibacter phosphatis	2	2	768
Actinobacteria	Actinobacteria	Propionibacterium acnes	2	2	929
Proteobacteria	Betaproteobacteria	Achromobacter xylosoxidans	2	2	774
Proteobacteria	Gammaproteobacteria	Enterobacter cloacae	1	1	487
Proteobacteria	Betaproteobacteria	Bordetella bronchiseptica	1	1	40
Proteobacteria	Alphaproteobacteria	Caulobacter sp. K31	1	1	192
Proteobacteria	Gammaproteobacteria	Buchnera aphidicola	1	1	498
Bacteroidetes	Flavobacteria	Flavobacterium gelidilacus	1	1	409
Tenericutes	Mollicutes	Mycoplasma hominis	1	1	435
Tenericutes	Mollicutes	Mesoplasma florum	1	1	58
Firmicutes	Clostridia	Alkaliphilus oremlandii	1	1	479

Tenericutes	Mollicutes	Mycoplasma mycoides	1	1	572
Proteobacteria	Gammaproteobacteria	Xanthomonas albilineans	1	1	45
Tenericutes	Mollicutes	Mycoplasma leachii	1	1	538
Proteobacteria	Alphaproteobacteria	Azospirillum sp. B510	1	1	246
Tenericutes	Mollicutes	Mycoplasma fermentans	1	1	389
Proteobacteria	Betaproteobacteria	Burkholderia mallei	1	1	467
Proteobacteria	Alphaproteobacteria	Ochrobactrum anthropi	1	1	419
Tenericutes	Mollicutes	Mycoplasma bovis	1	1	448
Proteobacteria	Alphaproteobacteria	Dinoroseobacter shibae	1	1	472
Proteobacteria	Gammaproteobacteria	Pseudomonas stutzeri	1	1	461
Tenericutes	Mollicutes	Mycoplasma sp. 1220	1	1	508
Tenericutes	Mollicutes	Mycoplasma gypis	1	1	541
Proteobacteria	Alphaproteobacteria	Brevundimonas diminuta	1	1	358
Proteobacteria	Gammaproteobacteria	Serratia liquefaciens	1	1	245
Firmicutes	Bacilli	Lactobacillus johnsonii	1	1	522

d) ***Lepetodrilus*** **sp. nov**

Phyla list

Phyla	Reads, num	Contigs, num	Contigs, bp
Proteobacteria	75	67	27063
Bacteroidetes	29	28	10669
Firmicutes	7	7	2811
Fusobacteria	4	4	1676
Unknown bacteria	4	4	1994
Cyanobacteria	3	3	1377
Tenericutes	3	3	1081
Aquificae	1	1	366
Planctomycetes	1	1	427

Classes list

Phyla	Classes	Reads, num	Contigs, num	Contigs, bp
Proteobacteria	Epsilonproteobacteria	44	36	14612
Bacteroidetes	Flavobacteria	24	23	9178
Proteobacteria	Gammaproteobacteria	21	21	7957
Proteobacteria	Alphaproteobacteria	9	9	4039
Firmicutes	Bacilli	5	5	2104
	Unknown bacteria	4	4	1994
Fusobacteria	Fusobacteria	4	4	1676
Cyanobacteria		3	3	1377
Tenericutes	Mollicutes	3	3	1081
Bacteroidetes	Cytophagia	2	2	813
Firmicutes	Clostridia	2	2	707
Bacteroidetes		2	2	546
Planctomycetes	Planctomycetacia	1	1	427
Aquificae	Aquificae	1	1	366
Proteobacteria	Betaproteobacteria	1	1	455
Bacteroidetes	Sphingobacteria	1	1	132

Genera list

Phyla	Classes	Genera	Reads, num	Contigs, num	Contigs, bp
Proteobacteria	Epsilonproteobacteria	Arcobacter	27	19	7914
Proteobacteria	Gammaproteobacteria	Buchnera	9	9	4188
Proteobacteria	Epsilonproteobacteria	Campylobacter	8	8	3243
Bacteroidetes	Flavobacteria	Flavobacterium	6	5	2014
Proteobacteria	Gammaproteobacteria	Escherichia	4	4	647
Proteobacteria	Alphaproteobacteria	Sphingopyxis	4	4	1842

Bacteroidetes	Flavobacteria	Croceibacter	4	4	1558
Bacteroidetes	Flavobacteria	Lacinutrix	4	4	1302
Proteobacteria	Epsilonproteobacteria	Sulfurovum	4	4	1398
Proteobacteria	Alphaproteobacteria	Bartonella	4	4	1776
		Unknown bacteria	4	4	1994
Proteobacteria	Epsilonproteobacteria	Sulfurimonas	4	4	1733
Cyanobacteria		Prochlorococcus	3	3	1377
Bacteroidetes	Flavobacteria	Cellulophaga	3	3	1160
Bacteroidetes	Flavobacteria	Zunongwangia	2	2	985
Proteobacteria	Gammaproteobacteria	Shewanella	2	2	713
Tenericutes	Mollicutes	Spiroplasma	2	2	580
Bacteroidetes		Candidatus Amoebophilus	2	2	546
Proteobacteria	Gammaproteobacteria	Aliivibrio	2	2	734
Bacteroidetes	Flavobacteria	Krokinobacter	2	2	980
Fusobacteria	Fusobacteria	Fusobacterium	2	2	996
Firmicutes	Bacilli	Lactococcus	1	1	363
Proteobacteria	Epsilonproteobacteria	Helicobacter	1	1	324
Fusobacteria	Fusobacteria	Leptotrichia	1	1	333
Firmicutes	Bacilli	Lactobacillus	1	1	437
Bacteroidetes	Flavobacteria	Weeksella	1	1	572
Proteobacteria	Gammaproteobacteria	Vibrio	1	1	505
Proteobacteria	Gammaproteobacteria	Yersinia	1	1	290
Firmicutes	Clostridia	Clostridium	1	1	364
Firmicutes	Clostridia	Caldicellulosiruptor	1	1	343
Firmicutes	Bacilli	Lysinibacillus	1	1	413
Bacteroidetes	Cytophagia	Marivirga	1	1	450
Planctomycetes	Planctomycetacia	Candidatus Kuenenia	1	1	427
Bacteroidetes	Flavobacteria	Fluviicola	1	1	476
Proteobacteria	Gammaproteobacteria	Thiomicrospira	1	1	411
Tenericutes	Mollicutes	Mesoplasma	1	1	501
Proteobacteria	Alphaproteobacteria	Orientia	1	1	421
Firmicutes	Bacilli	Geobacillus	1	1	455
Firmicutes	Bacilli	Listeria	1	1	436
Fusobacteria	Fusobacteria	Psychrilyobacter	1	1	347
Bacteroidetes	Cytophagia	Runella	1	1	363
Proteobacteria	Betaproteobacteria	Burkholderia	1	1	455
Bacteroidetes	Sphingobacteria	Pedobacter	1	1	132
Bacteroidetes	Flavobacteria	Riemerella	1	1	131
Proteobacteria	Gammaproteobacteria	Psychromonas	1	1	469
Aquificae	Aquificae	Sulfurihydrogenibium	1		

				1	366

Species list

Phyla	Classes	Species	Reads, num	Contigs, num	Contigs, bp
Proteobacteria	Epsilonproteobacteria	Arcobacter butzleri	19	13	5449
Proteobacteria	Gammaproteobacteria	Buchnera aphidicola	9	9	4188
Proteobacteria	Epsilonproteobacteria	Arcobacter nitrofigilis	8	6	2465
Bacteroidetes	Flavobacteria	Flavobacterium psychrophilum	5	4	1709
Proteobacteria	Gammaproteobacteria	Escherichia coli	4	4	647
Bacteroidetes	Flavobacteria	Lacinutrix sp. 5H-3-7-4	4	4	1302
Proteobacteria	Alphaproteobacteria	Sphingopyxis alaskensis	4	4	1842
Bacteroidetes	Flavobacteria	Croceibacter atlanticus	4	4	1558
Proteobacteria	Epsilonproteobacteria	Sulfurovum sp. NBC37-1	4	4	1398
Proteobacteria	Epsilonproteobacteria	Campylobacter lari	3	3	1135
Proteobacteria	Epsilonproteobacteria	Sulfurimonas denitrificans	3	3	1233
Proteobacteria	Epsilonproteobacteria	Campylobacter jejuni	3	3	1213
Cyanobacteria		Prochlorococcus marinus	3	3	1377
Bacteroidetes	Flavobacteria	Cellulophaga algicola	3	3	1160
Bacteroidetes	Flavobacteria	Krokinobacter sp. 4H-3-7-5	2	2	980
Bacteroidetes	Flavobacteria	Zunongwangia profunda	2	2	985
		endosymbiotic eubacteria	2	2	1072
Proteobacteria	Alphaproteobacteria	Bartonella sp. AR 15-3	2	2	852
Fusobacteria	Fusobacteria	Fusobacterium nucleatum	2	2	996
Proteobacteria	Alphaproteobacteria	Bartonella quintana	2	2	924
Bacteroidetes		Candidatus Amoebophilus asiaticus	2	2	546
Proteobacteria	Gammaproteobacteria	Aliivibrio salmonicida	2	2	734
Firmicutes	Bacilli	Lactococcus lactis	1	1	363
Bacteroidetes	Flavobacteria	Flavobacterium johnsoniae	1	1	305
Proteobacteria	Epsilonproteobacteria	Helicobacter pylori	1	1	324
Proteobacteria	Gammaproteobacteria	Vibrio harveyi	1	1	505
Tenericutes	Mollicutes	Spiroplasma cantharicola	1	1	291
		uncultured bacterium	1	1	417
Proteobacteria	Gammaproteobacteria	Shewanella frigidimarina	1	1	289
Bacteroidetes	Flavobacteria	Weeksella virosa	1	1	572
Proteobacteria	Epsilonproteobacteria	Sulfurimonas autotrophica	1	1	500
Bacteroidetes	Cytophagia	Marivirga tractuosa	1	1	450
Firmicutes	Bacilli	Lactobacillus salivarius	1	1	437
Firmicutes	Clostridia	Clostridium tetani	1	1	364
Tenericutes	Mollicutes	Spiroplasma citri	1	1	289

Proteobacteria	Gammaproteobacteria	Yersinia pestis	1	1	290
Fusobacteria	Fusobacteria	Leptotrichia buccalis	1	1	333
Firmicutes	Bacilli	Lysinibacillus sphaericus	1	1	413
Firmicutes	Clostridia	Caldicellulosiruptor kronotskyensis	1	1	343
Proteobacteria	Gammaproteobacteria	Shewanella woodyi	1	1	424
Bacteroidetes	Flavobacteria	Fluviicola taffensis	1	1	476
Proteobacteria	Epsilonproteobacteria	Campylobacter hominis	1	1	460
Proteobacteria	Gammaproteobacteria	Thiomicrospira crunogena	1	1	411
Proteobacteria	Epsilonproteobacteria	Campylobacter concisus	1	1	435
Tenericutes	Mollicutes	Mesoplasma florum	1	1	501
Firmicutes	Bacilli	Geobacillus sp. WCH70	1	1	455
Proteobacteria	Alphaproteobacteria	Orientia tsutsugamushi	1	1	421
Firmicutes	Bacilli	Listeria welshimeri	1	1	436
Fusobacteria	Fusobacteria	Psychrilyobacter atlanticus	1	1	347
Bacteroidetes	Cytophagia	Runella slithyformis	1	1	363
Proteobacteria	Betaproteobacteria	Burkholderia sp. CCGE1002	1	1	455
Aquificae	Aquificae	Sulfurihydrogenibium sp. YO3AOP1	1	1	366
Bacteroidetes	Sphingobacteria	Pedobacter heparinus	1	1	132
Bacteroidetes	Flavobacteria	Riemerella anatipestifer	1	1	131
Proteobacteria	Gammaproteobacteria	Psychromonas ingrahamii	1	1	469
Planctomycetes	Planctomycetacia	Candidatus Kuenenia stuttgartiensis	1	1	427
		uncultured bacterium ARCTIC06_H_11	1	1	505
